# Supplementary material for: Molecular driving force of a small molecule-induced protein disorder-order transition
Source: Commun Chem. 2026 Jan 14;9:65. doi: 10.1038/s42004-025-01869-5 (PMC12867982; doi:10.1038/s42004-025-01869-5)
Supplement: Supplementary file 2 — Supporting Information [file 42004_2025_1869_MOESM2_ESM.pdf]

Supporting Information

Molecular Driving Force of a Small Molecule-Induced Protein Disorder-Order Transition

Cesar Mendoza-Martinez,[1†‡] Arun A. Gupta,[1†‡] Salomé Llabrés,[1†‡] Paul N. Barlow,[1] Julien Michel[1*]

1) EaStCHEM School of Chemistry, University of Edinburgh, David Brewster Road, Edinburgh, EH9 3FJ, United Kingdom.
† Present Addresses: C. M-M: Drug Discovery Unit, University of Dundee, United Kingdom; A. A.G: Nuffield Department of Medicine, Center for Immuno--Oncology, University of Oxford, Oxford OX3 7DQ, United Kingdom; S.L.: Faculty of Pharmacy and Food Science, Universitat de Barcelona, Spain.

‡ These authors contributed equally to this work
*Corresponding author: Julien Michel

Email: julien.michel@ed.ac.uk

Table of contents

Computational protocols 4
Equilibrium Molecular Dynamics simulations (MD). 4
Enhanced sampling protocols (aMD/US/vFEP). 4
Accelerated Molecular Dynamics. 5
Definition of Collective Variables. 5
Umbrella Sampling. 5
2D variational Free Energy Profile (vFEP). 6
Analyses of the conformational ensembles. 6
Experimental protocols 6
Site-directed Mutant Constructs. 6
Protein Overproduction and Purification. 7
Isothermal Titration Calorimetry. 7
NMR Assignments. 8
NMR Titrations. 9
NMR Relaxation Measurements. 9
Figures 11
Fig. S1. SDS-PAGE profile for recombinant expressed WT and I19G mutant proteins across the purification cycle 11
Fig. S2. Mass spectrometry analysis of WT and ITC titration profiles with AM-7209. 12
Fig. S3. Mass spectrometry analysis of T16G mutant and ITC titration profiles with AM-7209. 13
Fig. S4. Mass spectrometry analysis of V14G mutant and ITC titration profiles with AM-7209. 14
Fig. S5. Mass spectrometry analysis of I19G mutant and ITC titration profiles with AM-7209. 15
Fig. S6. Mass spectrometry analysis of E23G mutant and ITC titration profiles with AM-7209. 16
Fig. S7. Mass spectrometry analysis of Q18G mutant and ITC titration profiles with AM-7209. 17
Fig. S8. Mass spectrometry analysis of T16GI19G double mutant and ITC titration profiles with AM-7209. 18
Fig. S9. Mass spectrometry analysis of E23R:R97E double mutant and ITC titration profiles with AM-7209. 19
Fig. S10. Mass spectrometry analysis of Δ6-16 MDM2 and ITC titration profiles with AM-7209. 20
Fig. S11. Assigned HSQC spectra of unliganded MDM2 WT and WT:AM-7209 complex at pH 7.0 and 288K. 21
Fig. S12. Assigned HSQC spectra of I19G mutant and I19G:AM-7209 complex at pH 7.0 and 288K. 22
Fig. S13. Comparison of WT apo (blue) and I19G HSQC apo (orange) spectra with specific residues showing large CSPs from "lid" region highlighted. 23
Fig. S14. Complex formation monitored for AM-7209 titration to ^{15}N uniformly labelled WT based on HSQC spectra 24

Fig. S15. Complex formation monitored for AM 7209 titration to ^{15}N uniformly labelled I19G mutant based on HSQC spectra. 25
Fig. S16. NMR analysis of AM-7209 interaction with WT and I19G mutant. 26
Fig. S17. NMR Chemical shift perturbations (CSPs) analysis of WT (apo) relative to I19G (apo) mutant. 28
Fig. S18. Comparison of NMR backbone dynamics for WT in Apo (WT) and AM7209-bound form (WT:AM-7209). 28
Fig. S19. Comparison of NMR backbone dynamics for I19G mutant in Apo and AM7209-bound form (I19G:AM-7209). 29
NMR relaxation rates R_1, R_2, and HetNOE were measured at pH 7.0 and 288K. Similar R_1, R_2 relaxation rates and HetNOE profiles were observed for residues (10-25) from "lid" region for I19G:AM-7209 bound form relative to I19G mutant in Apo form. 29
Fig. S20. RMSF calculations for double mutants. 30
Fig. S21. Definition of the collective variable used to describe the conformational preferences of the MDM2 lid dynamics. 31
Fig. S22. FES and Convergence plots for MDM2-WT. 32
Fig. S23. FES and Convergence plots for MDM2-WT:AM7209. 33
Fig. S24. FES and Convergence plots for MDM2-I19G. A) FES of the I19G MDM2 mutant (I19G). 34
Fig. S25. FES and Convergence plots for MDM2-I19G:AM7209. 35
Fig. S26. FES and Convergence plots for MDM2-WT:AM7209-Cl. 36
Fig. S27. Comparison between the experimental and predicted Chemical Shift Perturbation (CSP, ppm) between the I19G – WT proteins. 37
Fig S28. Analyses of the entropic changes in the conformational ensembles of the MDM2 systems. 38
Fig S29. Contact maps of the AM7209 bound systems. 39
Tables 40
Table S1. Accelerated Molecular Dynamics parameters used in the present work. 40
Table S2. List of 2D and 3D spectra used to assign the WT, WT:AM-7209, I19G and I19G:AM-7209 (spectral width; complex points and offset are indicated.) 41
Table S3. Parameters used in NMR backbone dynamics measurements 42
Table S4. Solvent Accessible Surface Area (SASA) of the conformational ensembles of MDM2. 43
Table S5. Details of the simulations included in the aMD/US/vFEP simulations included in this work. 43
Datasets 44
Dataset S1. (lid ensembles). 44
Dataset S2. (chemical shifts). 44
SI References 44

Computational protocols

Equilibrium Molecular Dynamics simulations (MD).

Proteins were parameterized with the general AmberFF99SBildn forcefield, solvated in a cubic box of TIP3P water molecules with the aid of the software FESetup.[1] This software generated inputs for simulations of complexes protein-ligand for wild type (aa. 6-125), mutants (V14G, V14D, V14T, T16G, Q18G, Q18E, I19G, I19A, I19V, I19E, E23G, E23L, E23Q, I19G:V14G, I19G:V14D, I19G:T16G, I19G:E23G, I19G:Q24G, V14G:T16G, T16G:Q24G, E23G:Q24G, E23R:R97E) and the truncated version of MDM2 (aa. 17-125). The starting point of every simulation was a conformation reported by Bueren-Calabuig et al.,[2] sampled from the lowest free energy region of a previous simulation of MDM2 in complex with the ligand PIP2. The ligand PIP2 was replaced with the ligand AM-7209. The crystal structure reported with AM-7209 (PDB ID: 4WT2) matched well with the chosen protein conformation.[3] Simulations were run with the SOMD engine. Every simulation was performed for 560 ns and trajectories analysed using the GROMACS software suite.[4] The helicity of residues 21 to 26 was estimated using the DSSP module, whereas RMSF values were computed using the g_rmsf module. Results were plotted with Matplotlib.

Enhanced sampling protocols (aMD/US/vFEP).

As starting points we employed three different structures of the MDM2 protein (residues 6-125) that differ in the conformation of the lid: semi-open state (obtained from model 2 of the NMR ensemble PDB id 1Z1M), closed state and ordered state described by Bueren-Calabuig et al. Missing residues on the C-terminal domain were modeled based on the model 2 of the NMR ensemble PDB id 2LZG (residues 1-125). The I19G mutant MDM2 initial models were built using the wild type models. The protein and ligand complexes were solvated on a triclinic TIP3P water box of 18 Å of radius and counterions were added to neutralize the systems.[5] The ff99SBildnnmr force field was used to model the protein and Joung and Cheatham parameters.[6,7] Nutlin-3a and AM-7209 ligands were parameterized using GAFF atom types and atomic charges derived by AM1-BCC method available within the *antechamber* tool in AMBER16 suite.[8,9,10]
Each system was energy minimized with a protocol combining 4500 steps of steepest descent followed by 4500 steps of conjugate gradient and subsequently smoothly equilibrated in a 6-ns long equilibration protocol. First, we heated the system from 100 K to 298 K over a 1ns-long simulation at constant volume with positional restraints on protein and ligand atoms (5 kcal $mol^{-1}Å^{-2}$). Then, each system was subjected to a smooth equilibration simulation (5 ns duration), while the restraints were gradually reduced for protein and ligand (decreasing by 1 kcal mol^{-1} $Å^{-2}$ per ns) at constant volume. Finally, the

density of the system was equilibrated by means of a 1 ns long simulation at 298 K in the NPT ensemble. Throughout the heating and equilibration stages was used for all bonds involving hydrogen atoms and a time step 2 fs was employed.[11]
Prior to the accelerated Molecular Dynamics (aMD) simulations,[12] a conventional 100 ns long simulation in the NPT ensemble, applying SHAKE to all bonds involving hydrogen atoms and using a 2 fs timestep, was performed per system using the GPU accelerated version of PMEMD from the AMBER16 software package.

Accelerated Molecular Dynamics.

aMD adds a positive energy boost to the potential energy function allowing the simulated systems to efficiently explore different regions of the Potential Energy Surface (PES) through reduced energy barriers. The potential boost ($\Delta V(r)$) is only applied when the average of the potential energy of the system ($V(r)$) is lower than a user defined energy reference (E_P).
A dual boost approach was used, with a potential energy boost applied to all the heavy atoms and an additional energy boost applied to all the torsions in the system. Therefore, the potential energy boost is given in **eq 1**.

$$\Delta V(r) = \frac{\left(E_P - V(r)\right)^2}{\left(\alpha_P + E_P - V(r)\right)} + \frac{\left(E_D - V_D(r)\right)^2}{\left(\alpha_D + E_D - V_D(r)\right)} \quad (1)$$

where E_P and E_D are the reference potential and torsional energies respectively; V_D is the dihedral component of the potential energy and α_P and α_D serve to control the intensity of the boost applied to each term. Initial E_P, E_D, α_P and α_D parameters were selected following the guidelines of Pierce and co-workers and based on preliminary MD runs. The values of E_P, E_D, α_P and α_D reported in **Table S1** were adopted for production runs after analysis of a series of preliminary MD runs.
To prevent that the applied energy boost drives the system towards high energy regions of the PES, which may result in unrealistic protein behavior like protein unfolding, the MDM2 core (25-125) was restrained using harmonic restraints with a force constant of 20 kcal mol^{-1} $Å^{-2}$ during the 300ns long aMD simulations.

Definition of Collective Variables.

Two suitable collective variables (CV) were used to describe the conformational preferences of the MDM2 lid. CV1 defines the extension of the lid as the distance between the Cα atoms of residues M6 and E23. CV2 accounts for the dihedral angle defined by the Cα atoms of residues D11, M50, M62 and V41 (lid-core dihedral angle). **Figure S21** depicts both CV.
The conformational space (CV space) defined by these two collective variables enabled to discriminate four different states for the lid conformation: open (CV2 > 220º), semi-open (CV1 < 24Å, 110º < CV2 < 220º), closed (CV1 > 24Å, 110º < CV2 < 220º) and ordered conformations (CV2 < 110º).

Umbrella Sampling.

To compute the equilibrium distribution of each of the lid conformational ensembles, we used the following US protocol.[13] The CV space was sampled within the CV1 values 5 - 45 Å with an interval of 2 Å and CV2 values 36 - 268 º with an interval of 8 º accounting for a total 524 bins per system. The initial coordinates were selected by choosing the closest snapshot to the average structure sampled for that bin in the aMD runs for each protein construct. The selected protein structures were extracted and used as the initial starting conformations for apo and ligand bound systems. Once ligands were added, protein and ligand-protein complexes were solvated again following the same protocol described in the system setup section. Prior to the US runs, the starting conformations were re-equilibrated using the same energy minimisation protocol and a brief equilibration protocol (500 ps of thermalisation and 500 ps equilibration in the NPT ensemble). Then, 4ns long production runs were performed using the same conditions described in the system setup section and applying harmonic potential restraints of 1 kcal $mol^{-1}Å^{-2}$ and 0.12 kcal $mol^{-1}deg^{-2}$ to maintain CV1 and CV2 respectively. These restraining force constants were chosen to avoid excessive energetic penalties for conformations slightly deviating from the target CV values. Values of the reaction coordinates were stored every 10 fs for post-processing.

2D variational Free Energy Profile (vFEP).

The 2D variational Free Energy Profile (vFEP) method was used to obtain unbiased free energy profiles along the defined CV space.[14] vFEP is a maximum likelihood parametric approach to reweight biased simulation data. To estimate uncertainties in free energies, all US trajectories were sub-divided into two parts of equal duration (2ns) and analyzed separately.

Analyses of the conformational ensembles.

Structural analysis of the conformational ensembles was performed using CPPTRAJ tool from the AmberTools23[15] and MDTraj.[16] The hydrophobic SASA was calculated with the Shrake & Rupley method implemented in MDTraj and weighted by the probabilities obtained from the FES surfaces. More sophisticated solvation entropy analyses using Grid Inhomogeneous solvation theory proved challenging owing to the difficulty of analysing solvent properties on a 3D grid surrounding a disordered protein region (data not shown). The prediction of chemical shifts was performed using the SHIFTX2 tool[17] and later scaled (exp_CSP = 0.052+2.035·comp_CSP, $R^2 = 0.707$) to deal with the underestimation of the CSPs from MD datasets.[18] The entropy changes between the conformational ensembles were calculated using the PDB2ENTROPY and the PDB2TRENT tools.[19] The conformational entropy was calculated using the presets (c=8 and k=5). The translational and rotational entropy of the AM-7209 ligand was calculated only considering the heavy atoms of the ligand.

Experimental protocols

Site-directed Mutant Constructs.

The human WT MDM2 (6-125 aa) and Δ6-16 MDM2 (17-125 aa) plasmid constructs were used for the current study as previously reported. The Molecular dynamics simulations previously discussed led us to design site-specific human MDM2 mutations. The human MDM2 (V14G, T16G, Q18G, I19G, E23G, T16G:I19G, E23R:R97E) single and double mutant constructs were custom gene-synthesized (GenArt) and sub-cloned into pET-20b expression vector (ampicillin-resistant), with a C-terminal hexahistidine-tag included after the protein sequence.

Protein Overproduction and Purification.

The human recombinant WT (6-125) and Δ6-16 MDM2 (17-125) proteins were over-expressed as previously published. The human MDM2 mutant constructs included V14G, T16G, Q18G, I19G, E23G, T16G:I19G with a C-terminal hexahistidine-tag. Recombinant proteins were overproduced in *Escherichia coli,* C41 (DE3) host cells for the WT, Δ1-16 MDM2, and BL21 (DE3) host cells for the single- and double-mutant MDM2 constructs used in the study. Overnight grown bacterial culture in LB broth medium with carbenicillin (100 mg/mL) were induced with 1.0 mM isopropyl-β-D-1-thiogalactopyranoside (IPTG) after reaching $O.D_{600}$ ~ 1.0 and further incubated at 25 °C for 4-6 hrs. Bacterial culture was centrifuged and cells was lysed twice in lysis buffer (20 mM Tris, 100 mM NaCl, 5 mM TCEP), pH 8.0 using Constant Cell Systems Disruptor (1.1kW TS Benchtop) set at 25 kpsi. The lysed fraction was centrifuged, and directly applied to a Ni^{2+}-affinity (GE Healthcare) column. Elution of C-terminal hexahistidine-tagged protein was achieved with a step gradient of 20 mM Tris, 100 mM NaCl, 100 mM imidazole, pH 8.0 buffer (by a ramp method from 8 to 70 % of the buffer mentioned). The final protein purification was achieved by pooling the Ni^{2+}-affinity column-eluted fractions and applying the concentrated samples onto a 16/60 Superdex75 size-exclusion chromatography column (GE Healthcare), pre-equilibrated with 20 mM phosphate, 50 mM NaCl, 1mM tris-(2-carboxyethyl) phosphine (TCEP), pH 7.0. The uniform ^{15}N-labelled and $^{13}C/^{15}N$-labelled MDM2 proteins were produced in *E. coli*, C41 (DE3) and BL21 (DE3) host cells for wild type and I19G mutant, respectively, grown in minimal medium (M9) containing $^{15}NH_4Cl$ (1.0 g/L) as nitrogen source and D-$^{13}C_6$-glucose (2.4 g/L) as carbon source, as previously described.[20] The purity of unlabeled and isotope labelled MDM2 proteins were initially confirmed by SDS-PAGE and further by Electrospray ionization time of flight mass spectrometric analysis. LC-MS analysis profiles of unlabeled WT and I19G MDM2 mutant are shown in **Figure S2A** and **S5A,** respectively.

Isothermal Titration Calorimetry.

ITC was used to measure the dissociation constant (K_D) of the MDM2 ligands Nutlin-3a and AM-7209. LB medium over-expressed proteins were used for this purpose. Nutlin-3a was purchased from commercial vendors and the AM-7209 sample was kindly provided by Amgen Inc. All titrations were performed using a MicroCal Auto-iTC200 isothermal titration calorimeter from Malvern Panalytical assuming one site of binding and the data were analyzed by using the MicroCal PEAQ-ITC Analysis Software version 1.1.0. The first test for every construct was made by using 20 injections of 2 µl ligand into protein, every 300 sec. The temperature in the cell was 25 ºC and stirring was done at 750 rpm. The

concentration of the protein in the cell was 10 μM and for ligands in the syringe 100 μM and 150 μM for Nutlin-3a and AM-7209, respectively. For WT MDM2 *versus* AM-7209 and MDM2-mutants protein versus AM-7209 ligand titrations, some dissociation constants were in the picomolar range and hence difficult to measure accurately by direct titration. Hence a competitive titration protocol was used instead in these cases. For the competitive titration protocol, a pre-mix of wild-type or mutant MDM2 proteins (10 μM) and Nutlin-3a (100 μM) was incubated for 2 hours and then the pre-formed MDM2-Nutlin-3a complex samples in the calorimeter cell were titrated with 150 μM AM-7209 in the syringe. Previous literature describes both compounds Nutlin-3a and AM-7209 bind to MDM2 with a 1: 1 stoichiometry.[3] Non-linear regression fitting routine was used with one binding site model equations, keeping n-value fixed as 1 to determine the best values of the fitting parameters KD and ΔH for all the direct and competitive ITC titration datasets as previously reported by Sigurskjold et al. The fitting routine is already implemented in the software package mentioned before.[21] K_D values and standard deviations were determined from an independent set of triplicate experiments for each of the titrations. ITC profiles for WT MDM2 and I19G MDM2 titrations with AM-7209 are shown in **Figure S2 and S5,** respectively.

NMR Assignments.

NMR spectra were collected on a Bruker Avance 800 MHz spectrometer with a cryogenic TCI probe at 15 °C, with protein samples in the buffer containing 20 mM phosphate, 50 mM NaCl, 1mM TCEP, (pH 7), 10% D_2O (v/v) and 0.01% TSP as an internal standard. Backbone sequential resonance assignment was achieved using a standard suite of triple resonance experiments[22] acquired on uniformly $^{13}C/^{15}N$-labelled samples of unliganded wild type WT, WT in complex with AM-7209 (WT:AM-7209), I19G mutant and I19G in complex with AM-7209 (I19G:AM7209). No significant decrease in sample stability for WT sample (Conc ~ 480 μM) was observed over a period of 5 days until experiments were completed. The NMR assignments for the unliganded WT (as shown in **Figure S11**) correlated well with the previous published assignments of $MDM2_{6\text{-}125}$ in the apo form in similar buffer conditions.[23] For the WT:AM-7209 and I19G:AM-7209 complex sample preparation, a starting dilute sample of the WT and I19G in complex with excess AM-7209 ligand (~ 1.2 molar ratio) with final DMSO concentration at 3 % (v/v) was concentrated to WT:AM-7209 (Conc ~ 1mM) and I19G:AM-7209 (Conc ~ 450μM), respectively. WT:AM-7209 and I19G:AM-7209 protein-ligand complexes samples were stable over the entire time course of the 3D data acquisition. Due to low protein sample stability of I19G mutant owing to the tendency of I19G mutant to aggregate and undergo rapid precipitation, especially at concentrations higher than 200 μM, 3D backbone experiments for I19G mutant were acquired at lower concentration (Conc ~ 135 μM).
Acquisition parameters for the experiments recorded on each of the four different samples of WT, WT:AM7209 (1:1.2 complex), I19G and I19G:AM7209 (ligand: protein molar ratio of 1.2:1) are summarized in **Table S2**. All triple resonance experiments were recorded in non-uniform sampling (NUS) mode with NUS of 25 % as implemented in Bruker Topspin software. The SMILE algorithm implemented in NMRPipe was used to reconstruct NUS data[5, 6] and subsequently analyzed using NMRFAM-Sparky and POKY.[24,25,26,27] For preliminary sequential NMR assignment, AUTOASSIGN was used.[28]

Further chemical shift assignment refinement was done using PINE-SPARKY.[29] NMR software packages used for raw NMR data processing and further analysis were made available through NMRbox cloud computing platform.[30] Random-coil chemical shifts were computed from POTENCI webserver[22] for WT MDM2 and the 19G mutant. Structural propensity was evaluated based on secondary $^{13}C^{\alpha}$ and ^{13}CO chemical shifts, determined for all four assigned datasets *i.e* WT, WT:AM7209, I19G, and I19G:AM7209, by comparing the assigned $^{13}C\alpha$ and ^{13}CO chemical shifts to random-coil values. Assigned backbone NMR chemical shifts for WT, WT:AM7209, I19G, and I19G:AM7209 have been deposited in Biological Magnetic Resonance Data Bank (BMRB) (entry accession numbers: 52216, 52219, 52220, 52221 respectively).

NMR Titrations.

Previous literature as well as recent published experimental calorimetric studies indicate AM-7209 bind to MDM2 in 1:1 equimolar stoichiometry $^{1}H,^{15}N$-HSQC measurements on the ^{15}N-labeled MDM2 proteins (WT and I19G mutant) were carried out in buffer containing 20 mM phosphate, 50 mM NaCl, 1mM TCEP, (pH 7), 10% D_2O (v/v) and 0.01% TSP as an internal standard. A sample of ^{15}N-labeled WT protein (50 μM) was titrated in seven stepwise increments of 0.06, 0.12, 0.24, 0.36, 0.48, 0.72 and final saturating molar ratio value of 1.2:1 ligand:protein with AM-7209 (300 μM stock concentration). For all the titration points, the final DMSO concentration was maintained at 3 % (v/v) within the protein. In similar fashion, ^{15}N-labeled I19G protein (50 μM) was titrated in five stepwise increments of 0.12, 0.24, 0.36, 0.48, 0.60 and final saturating molar ratio value of 1.2:1 ligand:protein. AM-7209 binding for WT and I19G mutant was monitored through the chemical shift perturbations of the backbone amides, recording a series of $^{1}H,^{15}N$-HSQC spectra on the 800 MHz spectrometer, with 256 (*t1*) X 2048 (*t2*) complex. A total of eight scans were acquired per time-increment and the recycle delay was set to 1.4 s.
Combined weighted ^{1}H and ^{15}N CSPs (Δω) were calculated using the **eq 2** below.[31]

$$\Delta\omega = \sqrt{\Delta\ ^{1}H^{2} + (0.154\Delta^{15}N)^{2}} \qquad (2)$$

where, $\Delta^{1}H$ and $\Delta^{15}N$ are the changes in the proton and nitrogen chemical shifts (in ppm), respectively. **Figure S16** shows compiled plotted results from CSPs analysis for AM-7209 interaction with WT MDM2 and the I19G mutant.

NMR Relaxation Measurements.

Backbone ^{15}N longitudinal (R_1) and ^{15}N transverse (R_2) relaxation measurements were acquired for uniform ^{15}N-labelled samples of WT (Conc ~ 370μM), WT:AM-7209 (Conc ~ 575 μM, 1:1.2 protein:ligand molar ratio), I19G (Conc ~ 175μM protein) and I19G:AM-7209 (~ 275 μM, 1:1.2 protein:ligand molar ratio) respectively in the buffer described above in the "NMR Assignments" section using previously described pulse sequences in an interleaved pseudo-3D manner, on the 800 MHz spectrometer at 15 °C. R_1 and R_2 measurements with eight different relaxation delays from 50 to 1500 ms and 16.9 to 186 ms, respectively (**see Table SI 3)** were employed with recycle delay set to 1.5 sec between the scans.[32] Processed 2D $^{1}H,^{15}N$ HSQC datasets for R_1 and R_2 measurements at different

relaxation delays were fitted using single exponentials within the PINT program.[33] The triplicate set of steady-state {^{1}H}-^{15}N heteronuclear (Het)NOE measurements were recorded at 800 MHz using a water flip-back NOE pulse sequence in an interleaved fashion to compute the uncertainties within the measured HetNOE values. Two-dimensionals datasets were processed using NMRpipe and HetNOE values were calculated by taking the ratio of peak intensities according to the equation I_{on}/I_{off} from experiments performed with and without ^{1}H pre-saturation.

Figures

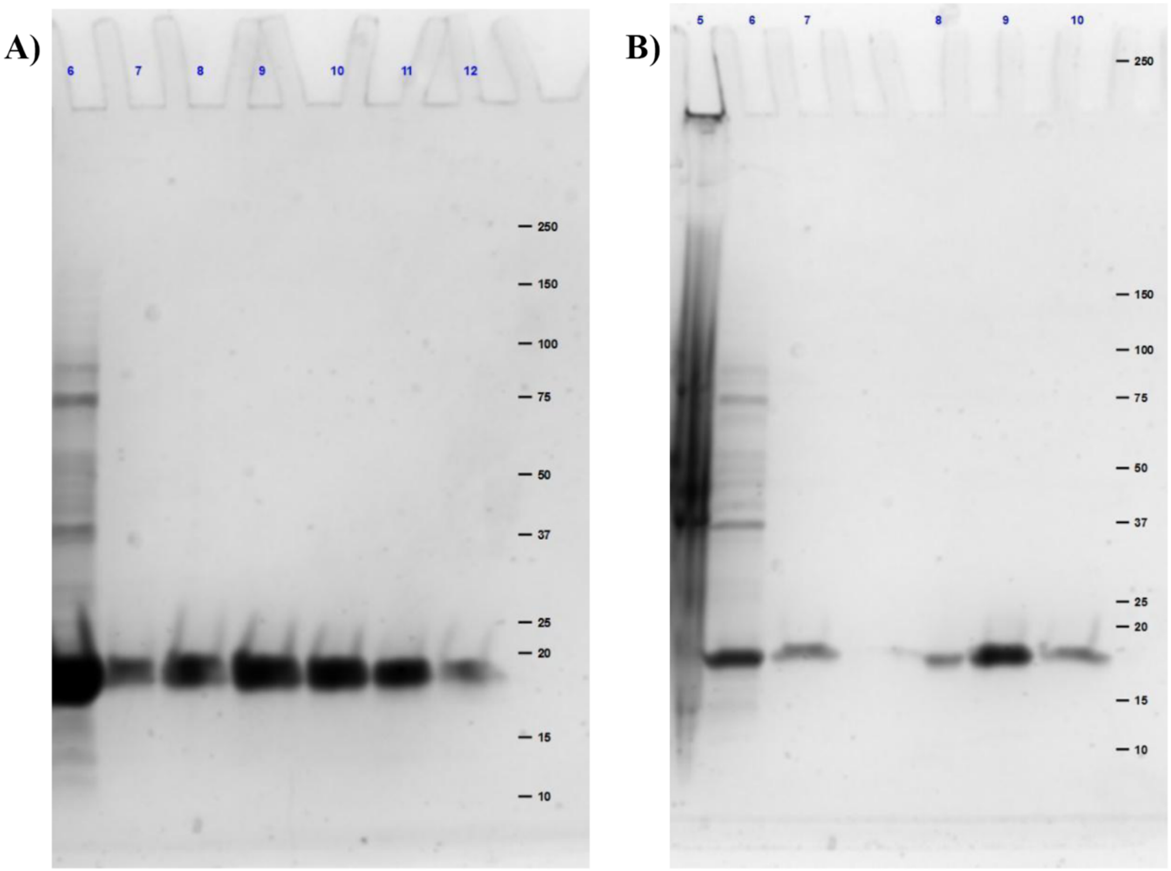

Fig. S1. SDS-PAGE profile for recombinant expressed WT and I19G mutant proteins across the purification cycle. (A) 15% SDS-PAGE gel analysis of recombinant expression of WT in C41(DE3) cells and various purification steps fraction (from left to right), Lane 6: supernatant fraction; Lane 7-12: Ni^{2+}-affinity column eluted fractions; the rightmost lane represents the protein molecular weight marker. (B) 15% SDS-PAGE gel analysis of recombinant expression of I19G mutant in BL21(DE3) cells and various purification steps fraction (from left to right), Lane 6: supernatant fraction; Lane 7: Ni^{2+}-affinity column eluted fraction; Lane 8-10: Size exclusion chromatography column eluted fractions; the rightmost lane represents the protein molecular weight marker.

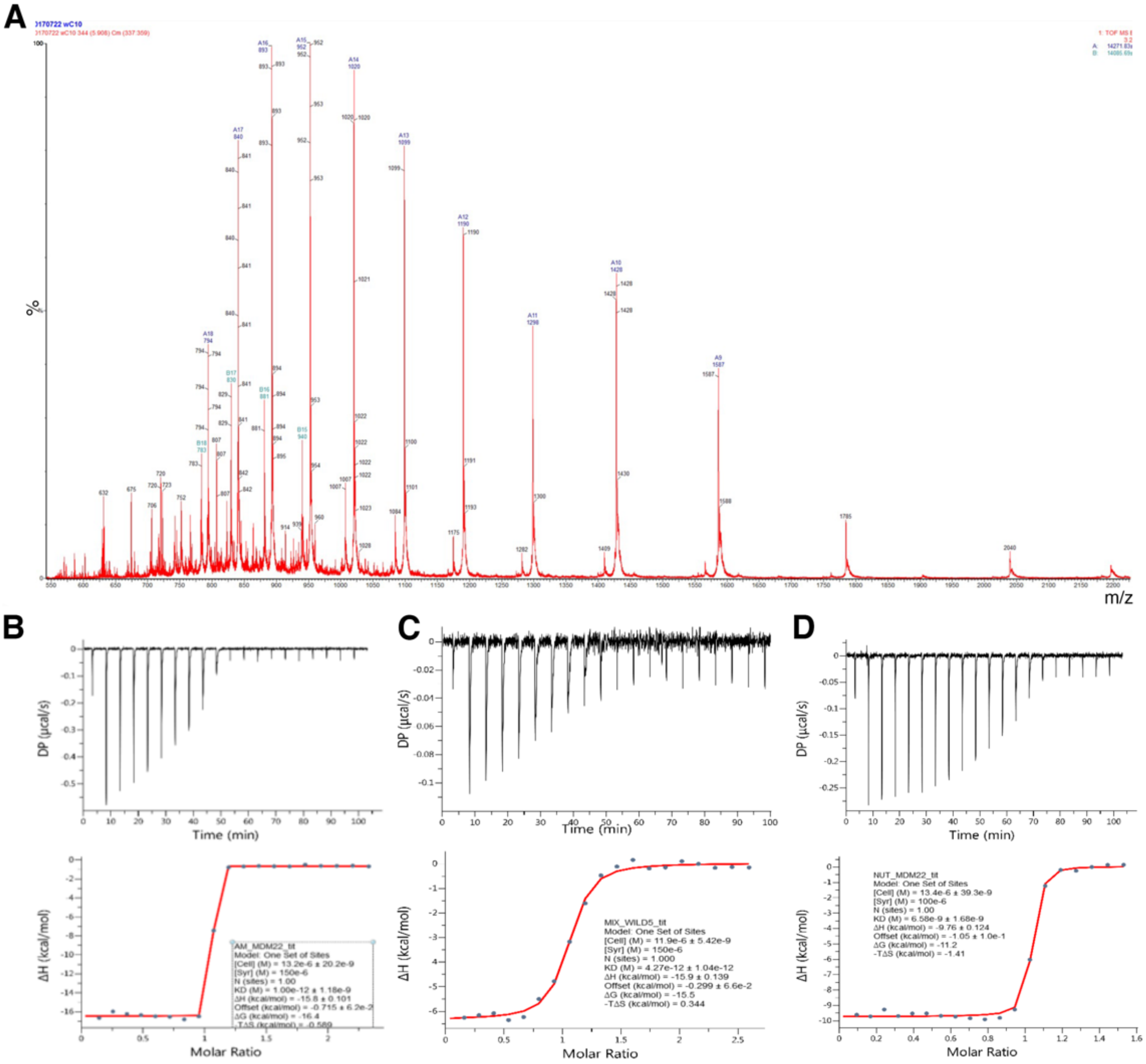

Fig. S2. Mass spectrometry analysis of WT and ITC titration profiles with AM-7209. **A**) Positive mode mass spectrum acquired by LC-MS for WT, deconvoluted average mass was calculated to be 14271.83Da, which is consistent with the N-terminal methionine cleaved-WT calculated theoretical average mass. **B**) AM-7209 direct ITC titration isotherm, **C**) AM-7209 competitive titration isotherm with Nutlin3a-WT pre-mixed complex, **D**) Nutlin-3a direct ITC titration isotherm with WT.

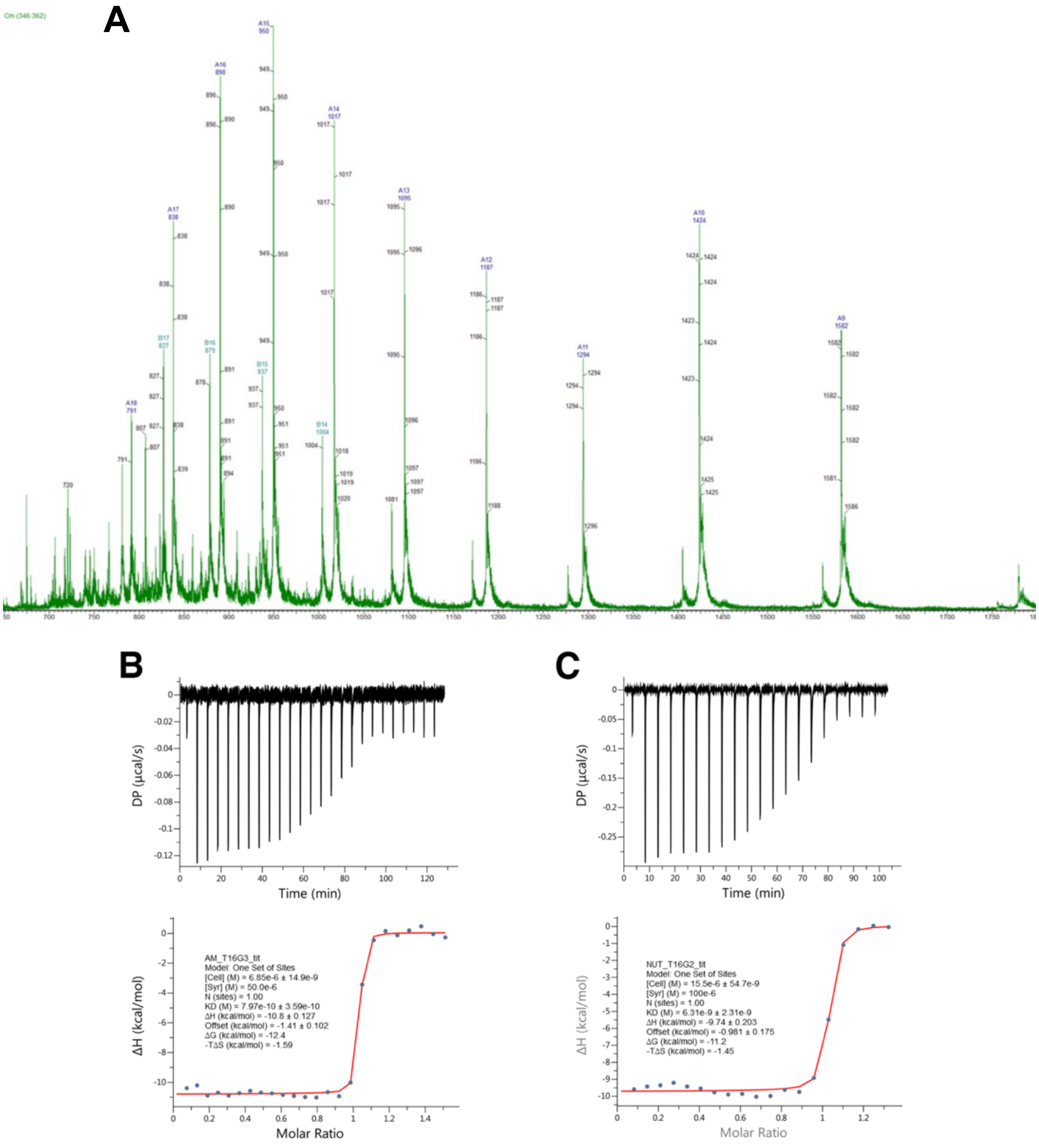

Fig. S3. Mass spectrometry analysis of T16G mutant and ITC titration profiles with AM-7209. A) Positive mode mass spectrum acquired by LC-MS for the T16G mutant, deconvoluted average mass was calculated to be 14228.45 Da, which is consistent with the N-terminal methionine cleaved T16G mutant calculated theoretical average mass. B) AM-7209 direct ITC titration isotherm with T16G mutant, C) Nutlin-3a direct ITC titration isotherm with T16G mutant.

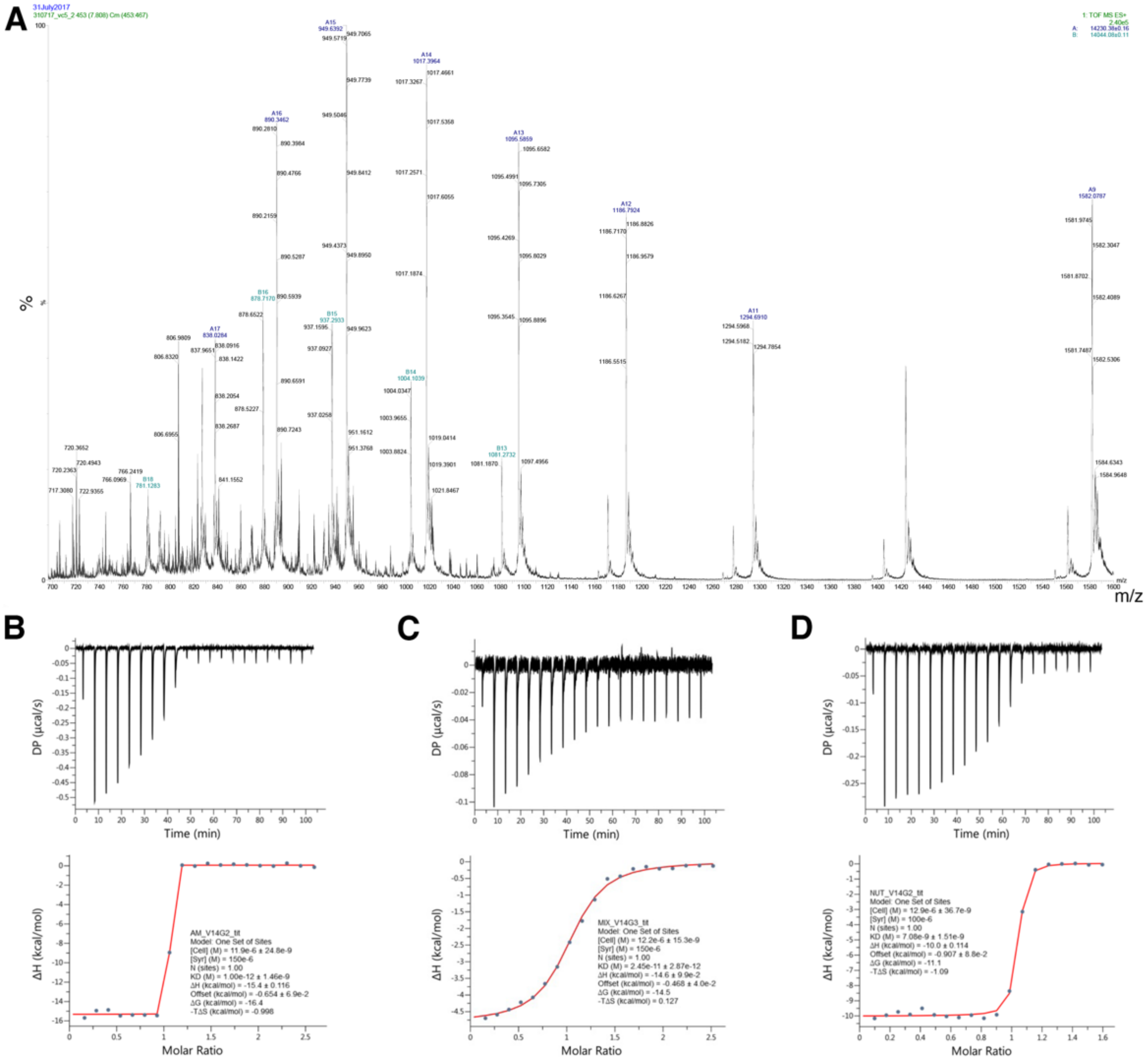

Fig. S4. Mass spectrometry analysis of V14G mutant and ITC titration profiles with AM-7209. A) Positive mode mass spectrum acquired by LC-MS for the V14G mutant, deconvoluted average mass was calculated to be 14230.38 Da, which is consistent with the N-terminal methionine cleaved V14G calculated theoretical average mass. B) AM-7209 direct ITC titration isotherm, C) AM-7209 competitive titration isotherm with Nutlin3a-V14G mutant pre-mixed complex, D) Nutlin-3a direct ITC titration isotherm with V14G mutant.

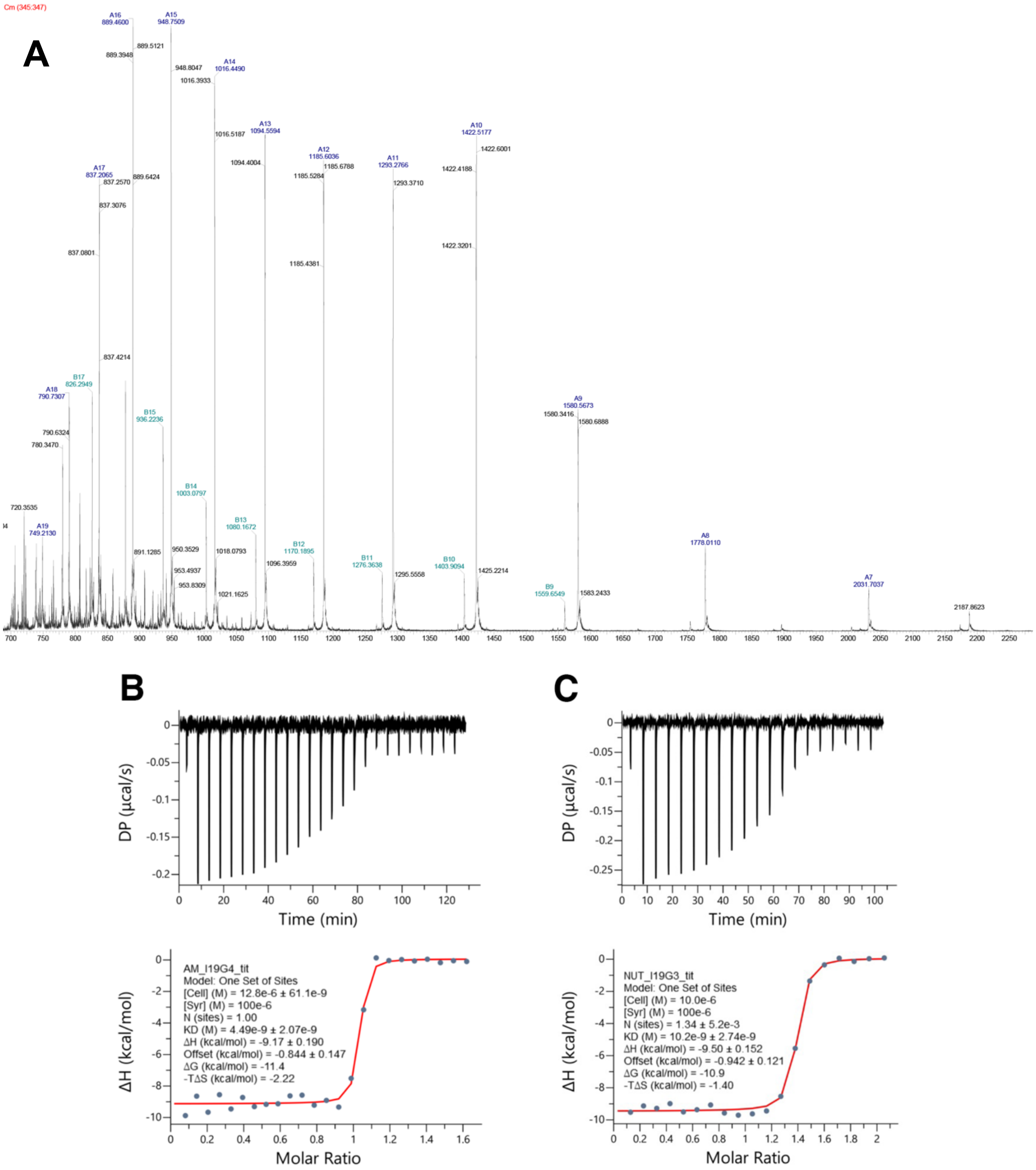

Fig. S5. Mass spectrometry analysis of I19G mutant and ITC titration profiles with AM-7209. A) Positive mode mass spectrum acquired by LC-MS for the I19G mutant, deconvoluted average mass was calculated to be 14215.73 Da, which is consistent with the N-terminal methionine cleaved I19G calculated theoretical average mass. B) AM-7209 direct ITC titration isotherm with I19G mutant, C) Nutlin-3a direct ITC titration isotherm with I19G mutant.

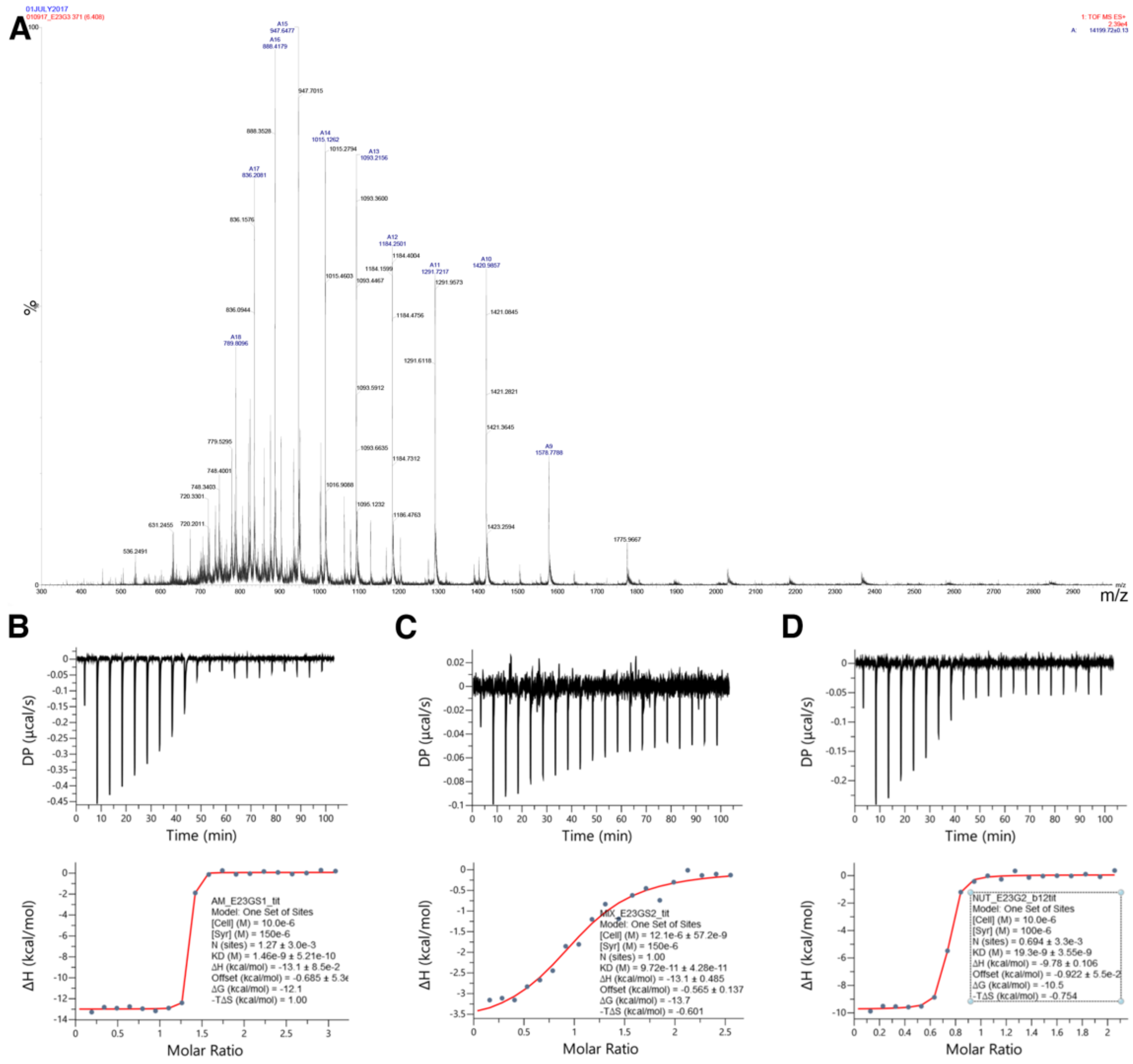

Fig. S6. Mass spectrometry analysis of E23G mutant and ITC titration profiles with AM-7209. A) Positive mode mass spectrum acquired by LC-MS for the E23G mutant, deconvoluted average mass was calculated to be 14199.72 Da, which is consistent with the N-terminal methionine cleaved E23G calculated theoretical average mass. B) AM-7209 direct ITC titration isotherm, C) AM-7209 competitive titration isotherm with Nutlin3a-E23G mutant pre-mixed complex, D) Nutlin-3a direct ITC titration isotherm with E23G mutant.

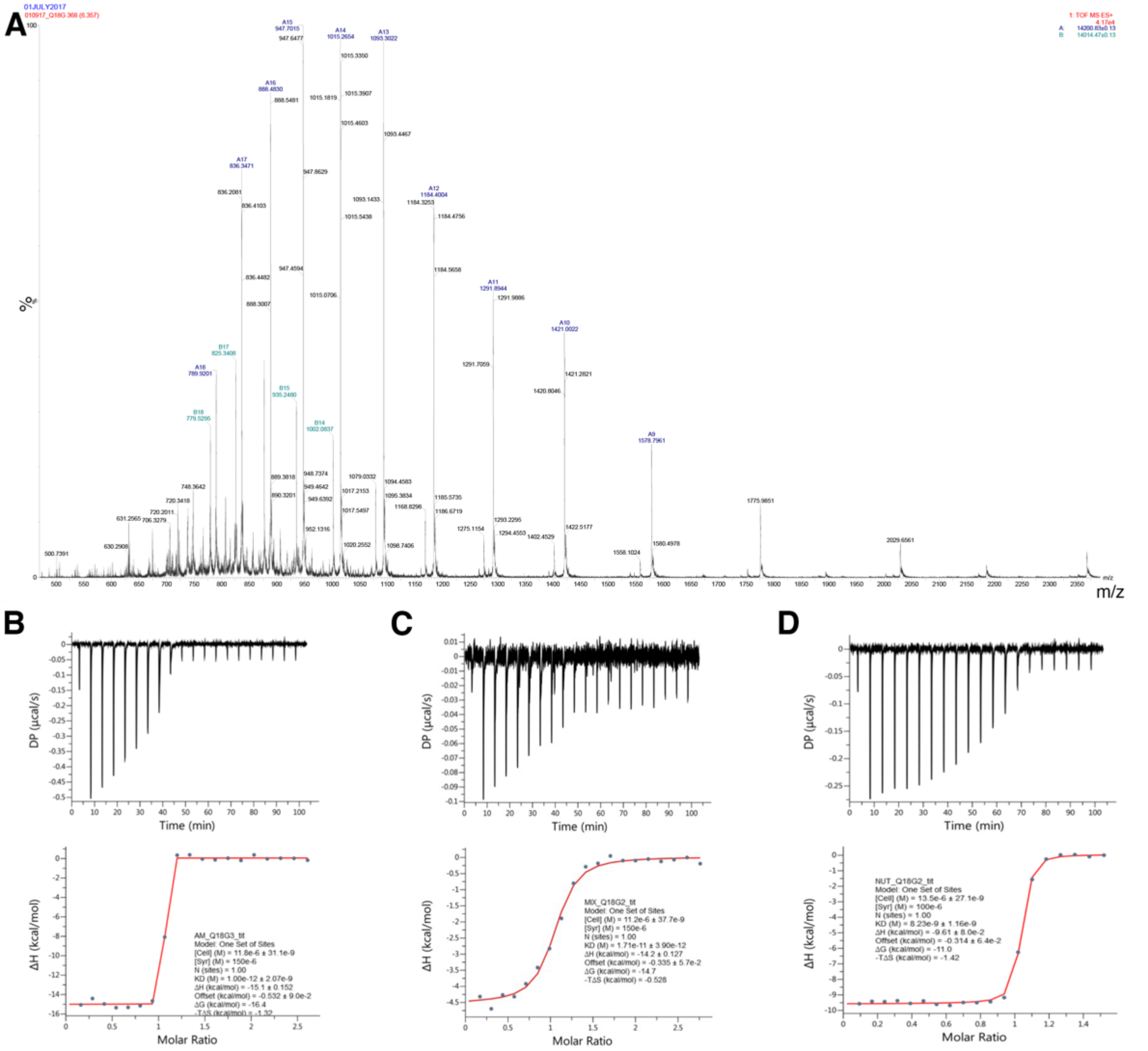

Fig. S7. Mass spectrometry analysis of Q18G mutant and ITC titration profiles with AM-7209. A) Positive mode mass spectrum acquired by LC-MS for the Q18G mutant, deconvoluted average mass was calculated to be 14200.83 Da, which is consistent with the N-terminal methionine cleaved Q18G calculated theoretical average mass. B) AM-7209 direct ITC titration isotherm, C) AM-7209 competitive titration isotherm with Nutlin3a-Q18G mutant pre-mixed complex, D) Nutlin-3a direct ITC titration isotherm with Q18G mutant.

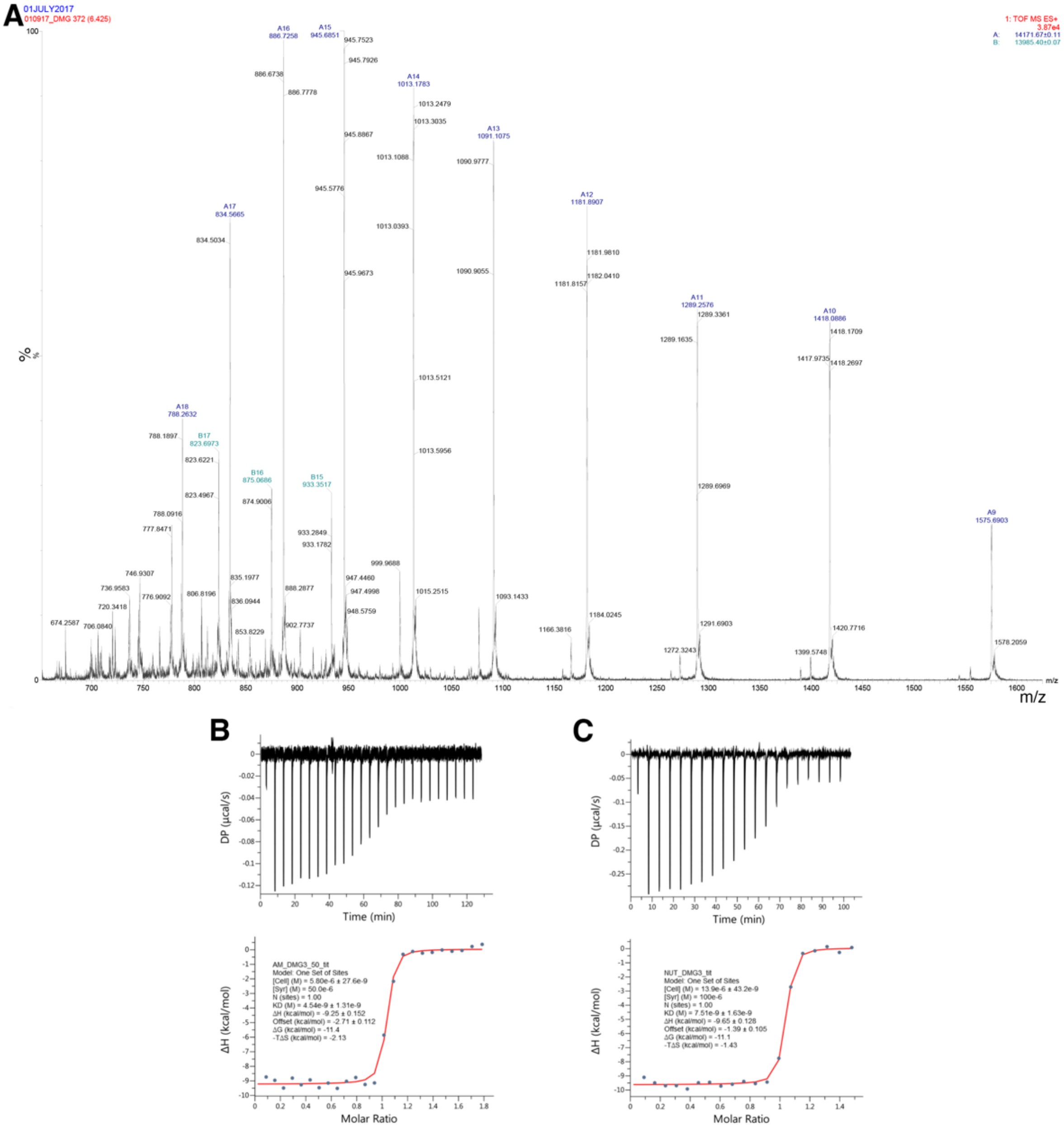

Fig. S8. Mass spectrometry analysis of T16GI19G double mutant and ITC titration profiles with AM-7209. A) Positive mode mass spectrum acquired by LC-MS for the T16G:I19G mutant, deconvoluted average mass was calculated to be 14171.67 Da, which is consistent with the N-terminal methionine cleaved T16G:I19G calculated theoretical average mass. B) AM-7209 direct ITC titration isotherm with T16G:I19G mutant, C) Nutlin-3a direct ITC titration isotherm with T16G:I19G mutant.

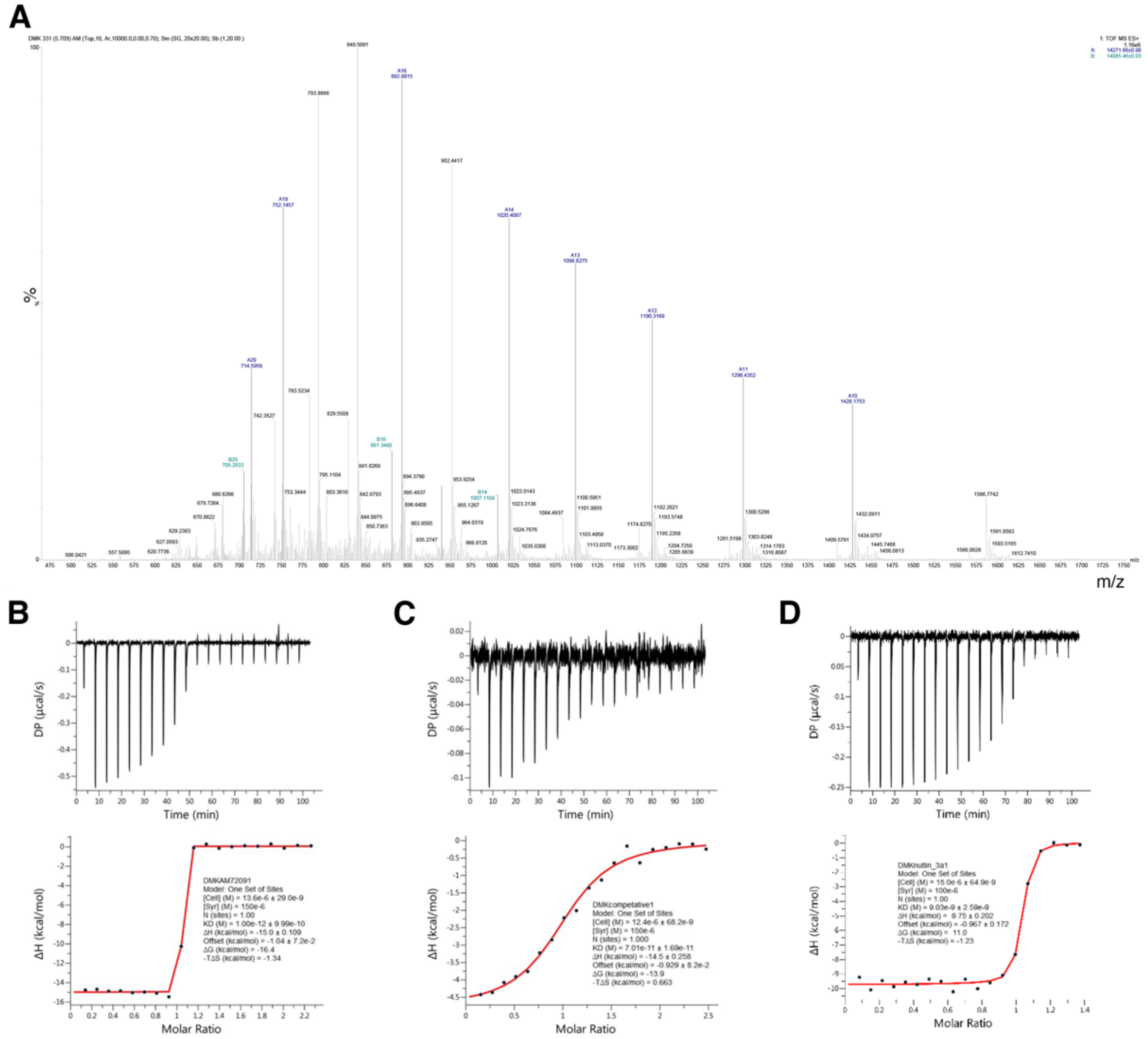

Fig. S9. Mass spectrometry analysis of E23R:R97E double mutant and ITC titration profiles with AM-7209. A) Positive mode mass spectrum acquired by LC-MS for the E23R:R97E mutant, deconvoluted average mass was calculated to be 14271.66 Da, which is consistent with the N-terminal methionine cleaved E23R:R97E calculated theoretical average mass. B) AM-7209 direct ITC titration isotherm, C) AM-7209 competitive titration isotherm with Nutlin3a- E23R:R97E mutant pre-mixed complex, D) Nutlin-3a direct ITC titration isotherm with E23R:R97E mutant.

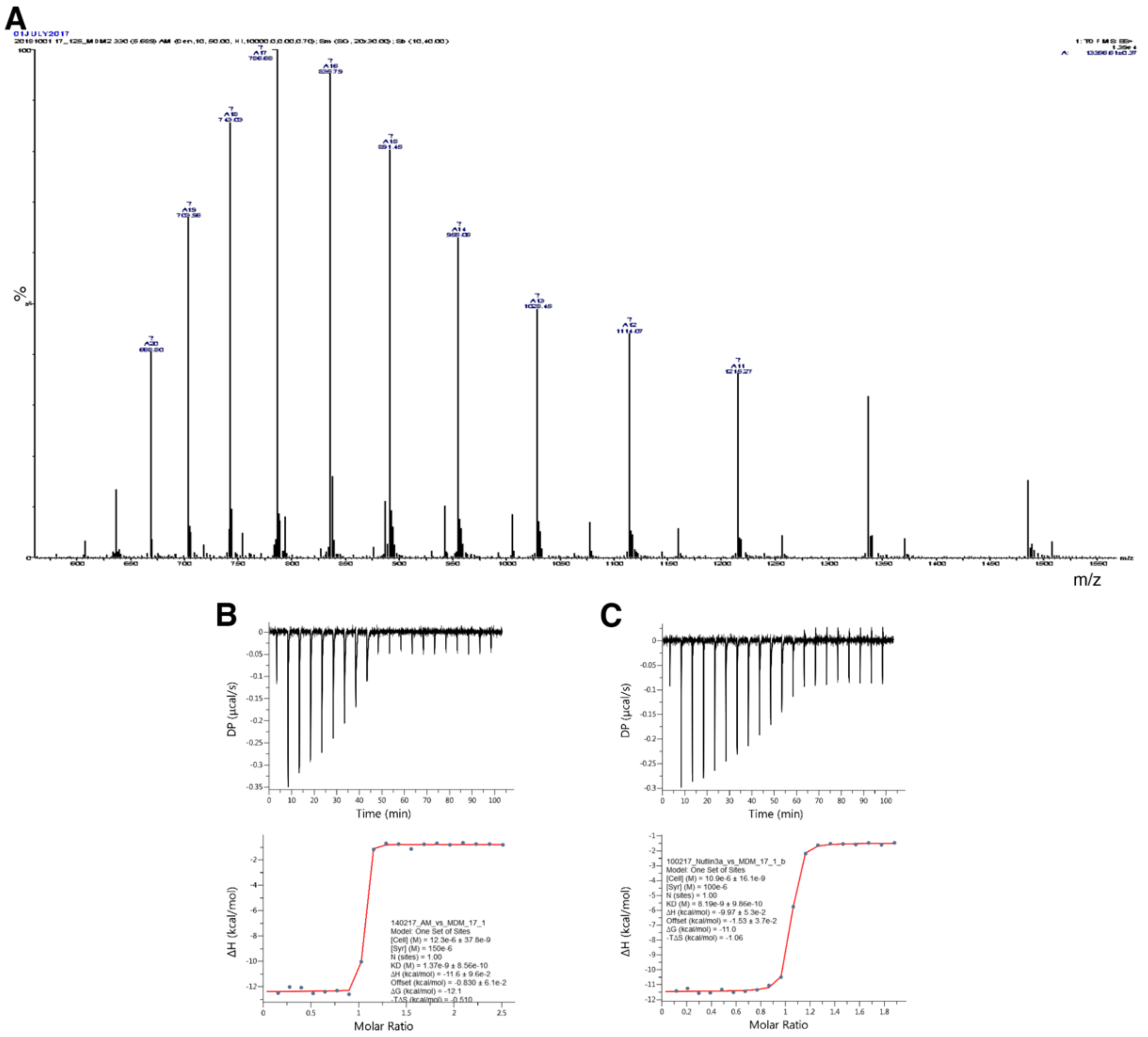

Fig. S10. Mass spectrometry analysis of Δ6-16 MDM2 and ITC titration profiles with AM-7209. A) Positive mode mass spectrum acquired by LC-MS for the Δ6-16 MDM2, deconvoluted average mass was calculated to be 13556.61Da, which is consistent with the N-terminal methionine cleaved Δ6-16 MDM2 calculated theoretical average mass. B) AM-7209 direct ITC titration isotherm with Δ6-16 MDM2, C) Nutlin-3a direct ITC titration isotherm with Δ6-16 MDM2.

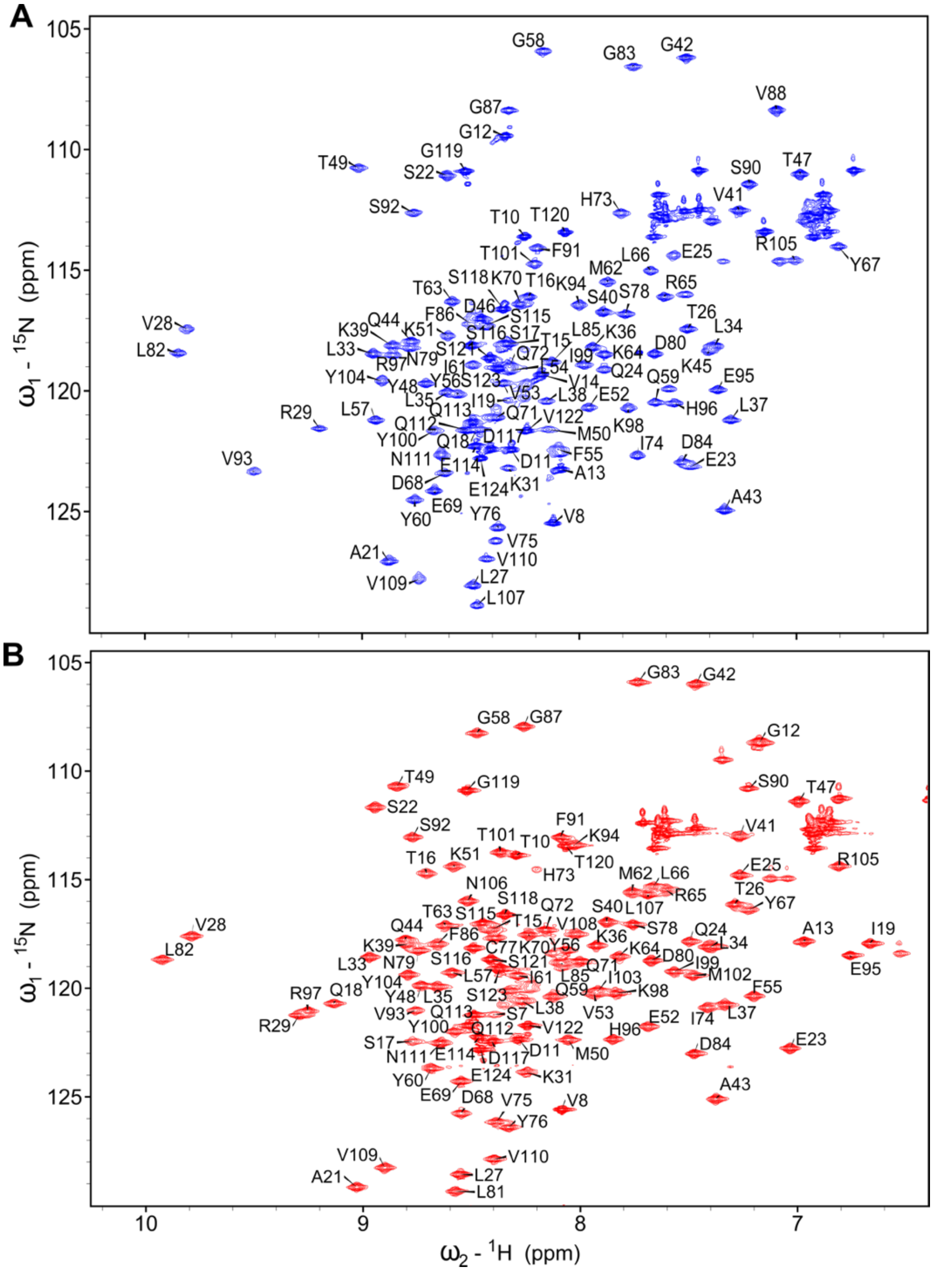

Fig. S11. Assigned HSQC spectra of unliganded MDM2 WT and WT:AM-7209 complex at pH 7.0 and 288K. Residue specific assignment of the backbone amide frequencies as a function of MDM2$_{(6\text{-}125)}$ protein sequence in (A) WT (blue) and (B) WT:AM-7209 (red). More than 91% of the backbone resonances were assigned for unliganded MDM2 WT and near complete >96% of the backbone for WT:AM-7209 complex (except prolines). Notably resonances for M6 and S7 residues are not observed in the "lid" region of the WT spectra. For WT:AM-7209 complex spectra, residues M6 and V14 were not observable from the "lid" region.

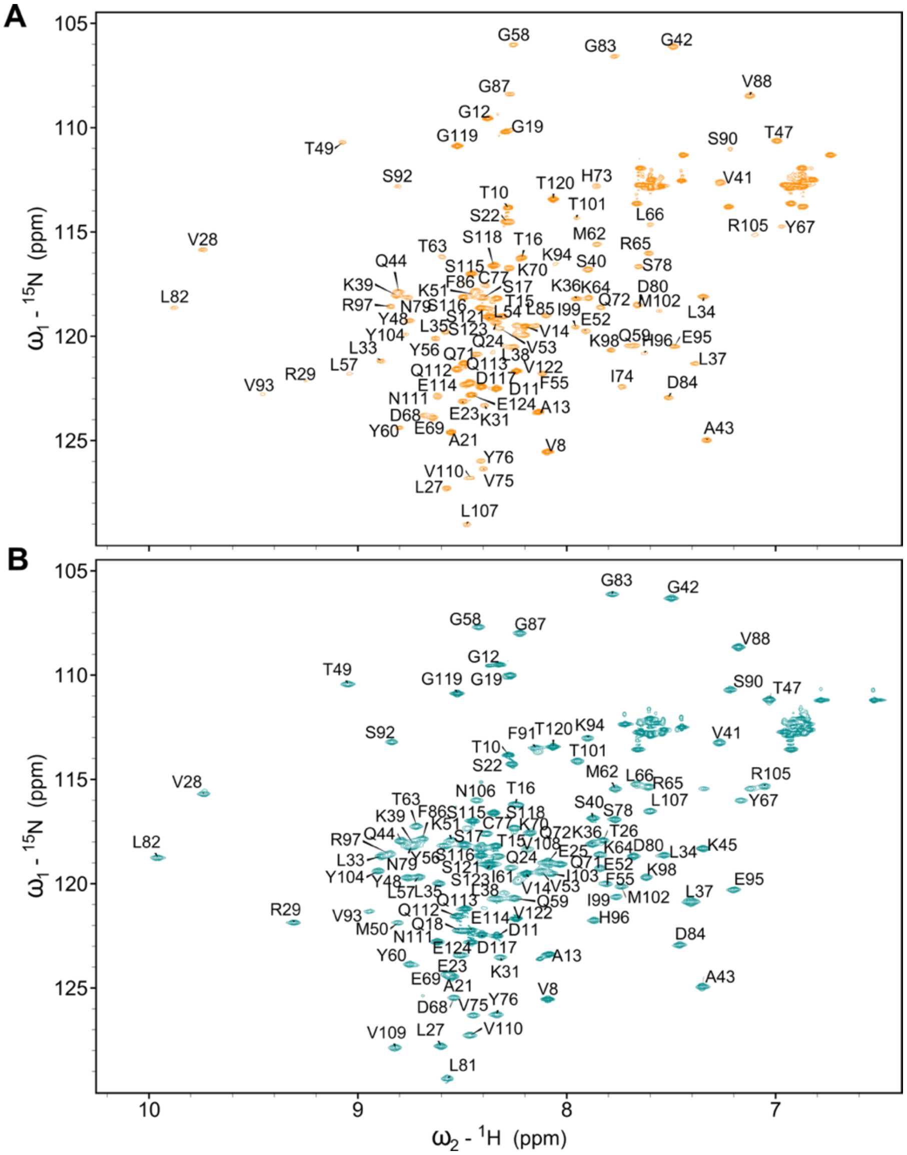

Fig. S12. Assigned HSQC spectra of I19G mutant and I19G:AM-7209 complex at pH 7.0 and 288K. Residue specific assignment of the backbone amide frequencies as a function of MDM2$_{(6\text{-}125)}$ protein sequence for (A) I19G (orange) and (B) I19G:AM-7209 (cyan). More than 75% of the backbone shifts were assigned for I19G mutant and >88% of the backbone shifts for I19G:AM-7209 complex (except prolines). Additional resonances for Q18, E25 and T26 were not observed in "lid" region for I19G spectra compared to WT. Also, several resonances from the C-terminal region are missing in the I19G spectra. For I19G:AM-7209 complex spectra, residues M6-V8 were not detected.

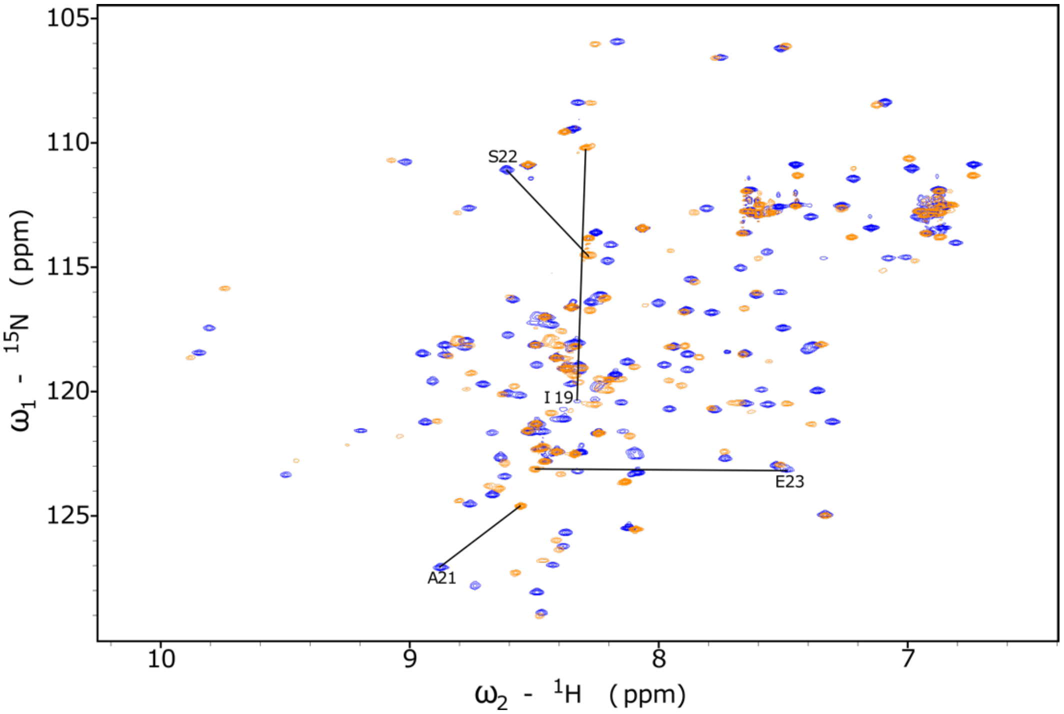

Fig. S13. Comparison of WT apo (blue) and I19G HSQC apo (orange) spectra with specific residues showing large CSPs from "lid" region highlighted. Most of the large CSPs are located in the immediate vicinity of residue 19, suggesting limited structural changes between WT and the I19G mutant in apo forms.

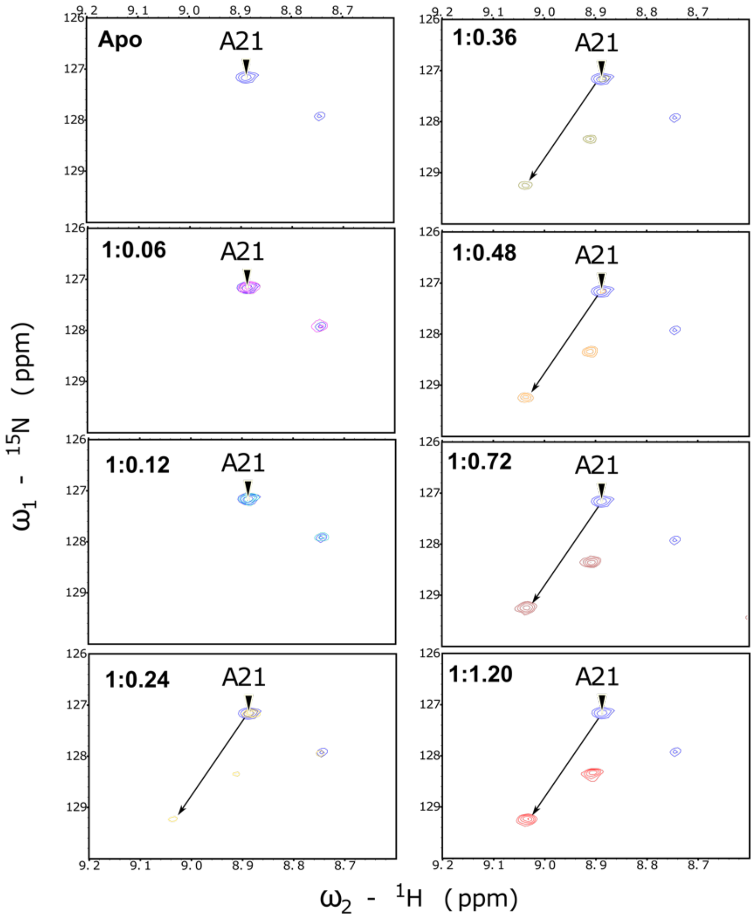

Fig. S14. Complex formation monitored for AM-7209 titration to ^{15}N uniformly labelled WT based on HSQC spectra. Overlaid ^{1}H, ^{15}N HSQC spectra showing chemical shift perturbations (CSPs) with lid region residue Ala-21 cross peak of WT highlighted is monitored upon binding to AM 7209. Two separate sets of cross peaks were observed in ^{1}H, ^{15}N HSQC spectra, one corresponding to apo and the other for WT bound to AM-7209 at sub-molar concentrations of 1:0.24, 1:0.36 and 1:0.48 (protein: ligand molar ratio). Complete disappearance of the apo Ala-21 cross peak and appearance of bound cross peak indicate exchange between WT apo (blue) and bound, 1:1.20 (red) resonances is in the slow exchange regime. The spectrum shows titrations up to a 1:1.20 complex (protein: ligand molar ratio) with WT Apo ^{1}H, ^{15}N HSQC spectra acquired at a starting concentration of 0.05mM and 288 K.

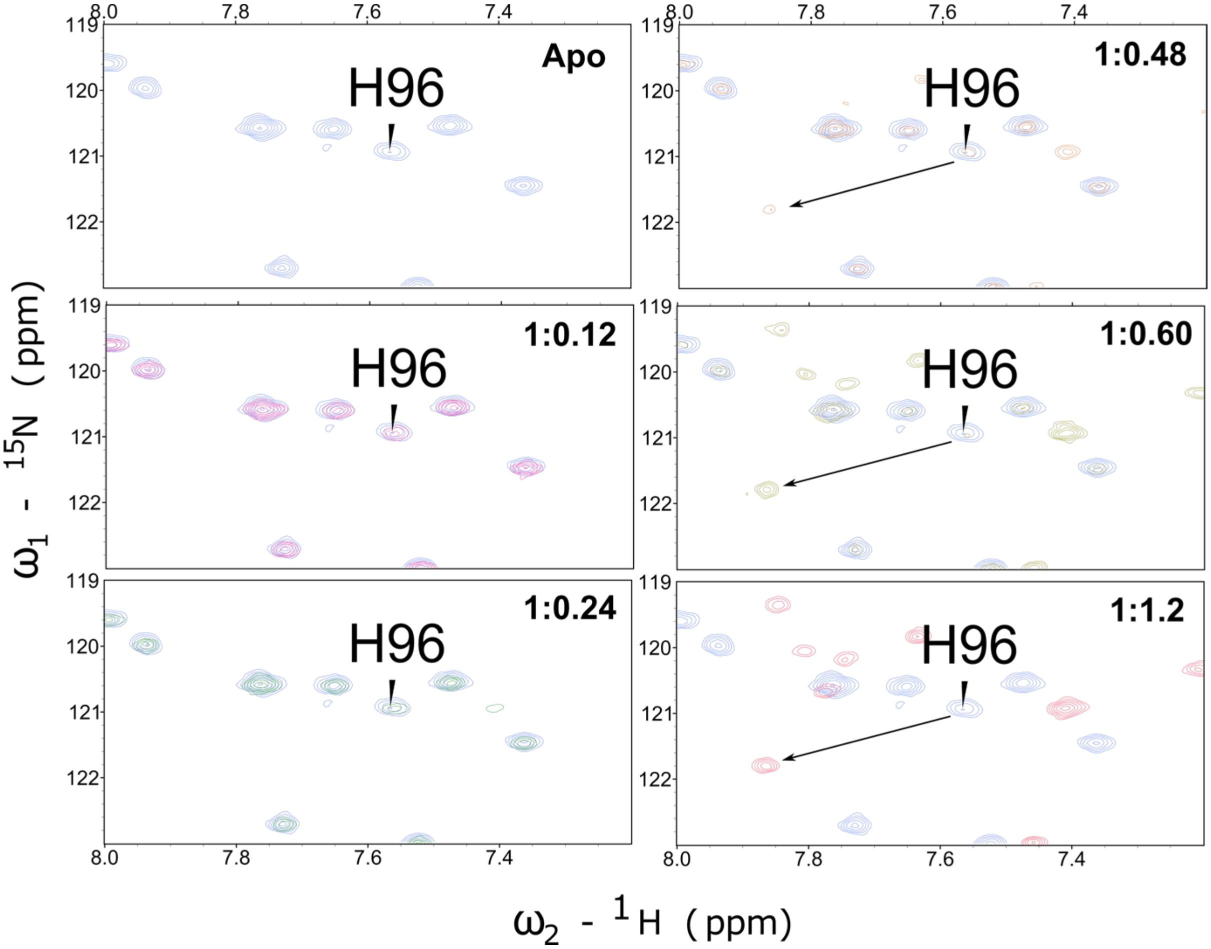

Fig. S15. Complex formation monitored for AM 7209 titration to ^{15}N uniformly labelled I19G mutant based on HSQC spectra. Overlaid ^{1}H, ^{15}N HSQC spectra showing chemical shift perturbations (CSPs) with core region residue His-96 cross peak of the I19G mutant highlighted is monitored upon binding of AM-7209. Two separate sets of cross peaks were observed in ^{1}H, ^{15}N HSQC spectra, one corresponding to apo (royal blue) and the other for I19G mutant bound to AM-7209 at sub-molar concentrations of 1:048 (chocolate) and 1:0.60 (olive). Complete disappearance of the apo His-96 cross peak and appearance of bound cross peak indicate exchange between I19G mutant apo (royal blue) and bound, 1:1.20 (crimson) resonances is in the slow exchange regime. The spectrum shows titrations up to a 1:1.20 complex (protein: ligand molar ratio) with I19G Apo ^{1}H, ^{15}N HSQC spectra acquired at a starting concentration of 0.05mM and 288 K.

A)

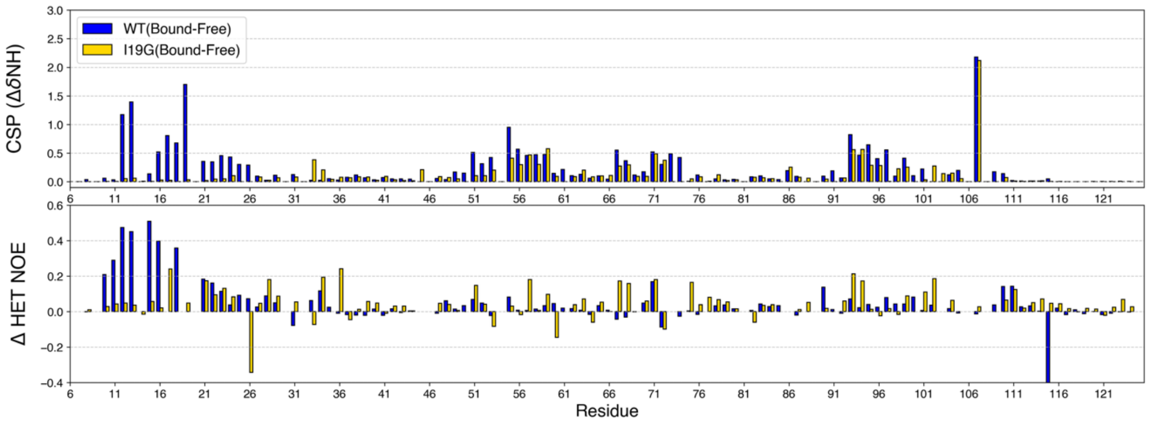

B)

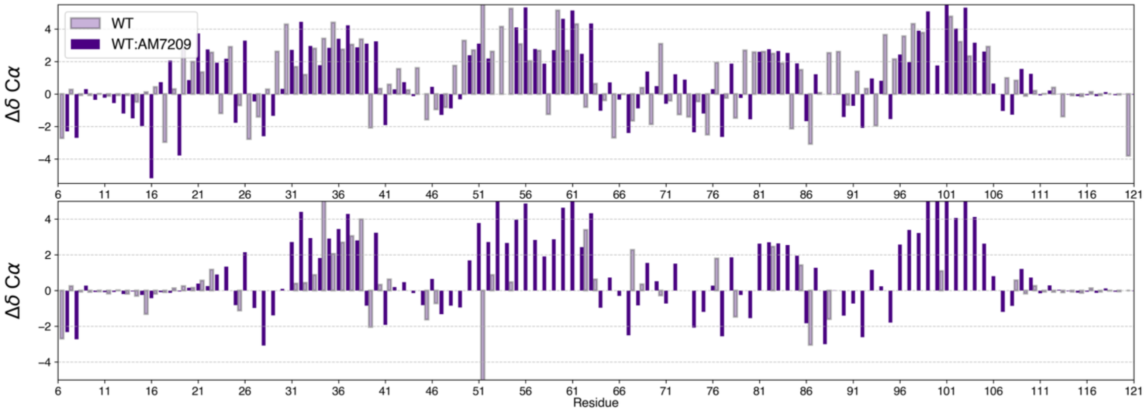

C)

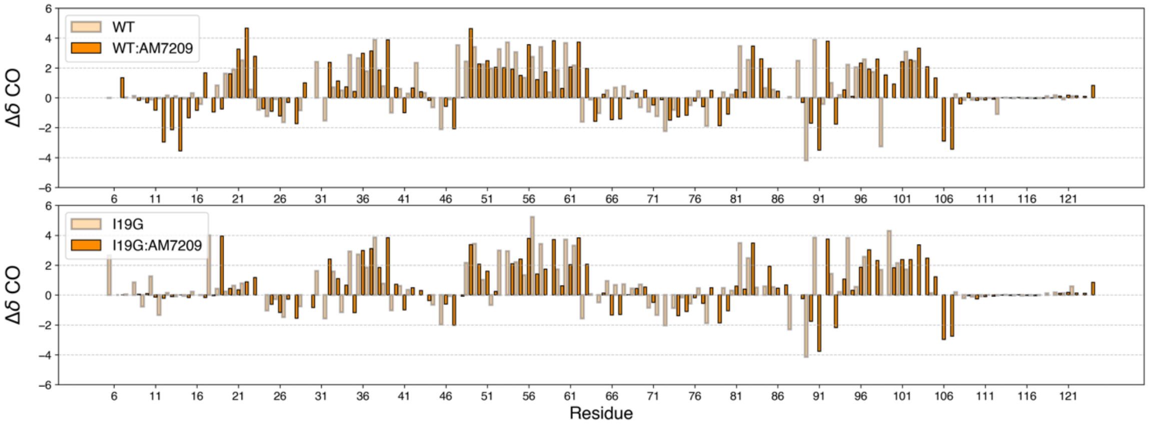

Fig. S16. NMR analysis of AM-7209 interaction with WT and I19G mutant. A) Chemical shift perturbations (CSP) and ΔHetNOE. Substantial chemical shifts changes with CSP values > 0.50 ppm (arbitrarily value selected) and strong positive deviations in ΔHetNOE indicate structural ordering within "lid" region for WT:AM-7209 in comparison to WT. No significant changes in CSPs and small positive deviations in ΔHetNOE values within "lid" region for I19G:AM-7209 complex with relative to I19G were observed. B) Residue specific secondary chemical shift (SCS) ΔδCα plotted as a function of MDM2 $_{(6-125)}$ protein sequence for WT and I19G in free and AM-7209 bound form. Further pronounced increase in propensity for β-strand (residues 10-16) and α-helical (residues 21-

24) secondary structure elements formation within “lid” region for WT:AM-7209 in comparison to WT is clearly indicated from SCS Cα. C) Residue specific secondary chemical shift (SCS) ΔδCO plotted as a function of $MDM2_{(6\text{-}125)}$ protein sequence for WT and I19G in free and AM-7209 bound form. Further pronounced increase in propensity for β-strand (residues 10-16) and α-helical (residues 21-24) secondary structure elements formation within “lid” region for WT:AM-7209 in comparison to WT is clearly indicated from SCS CO values.

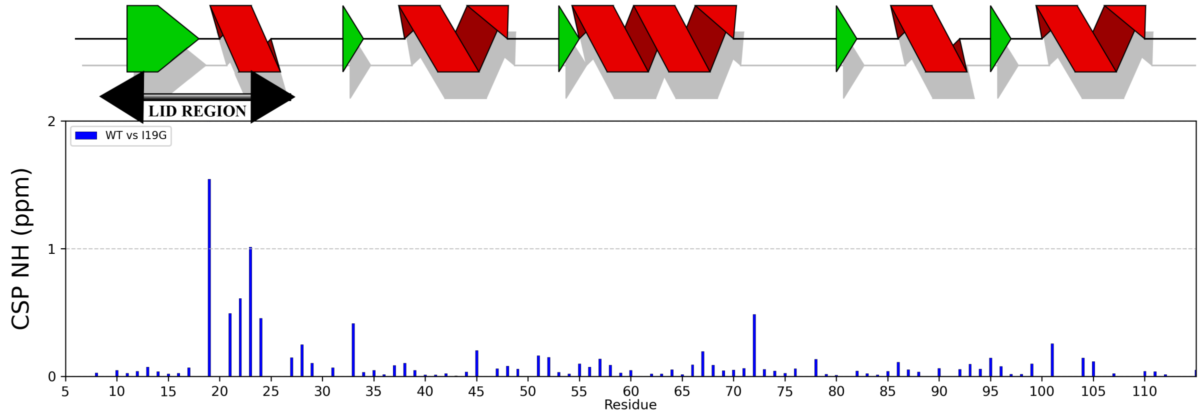

Fig. S17. NMR Chemical shift perturbations (CSPs) analysis of WT (apo) relative to I19G (apo) mutant. Substantial chemical shifts changes with CSP values > 0.5 ppm (arbitrarily value selected) within “lid” region (residues 6-25) for WT in comparison to I19G were observed.

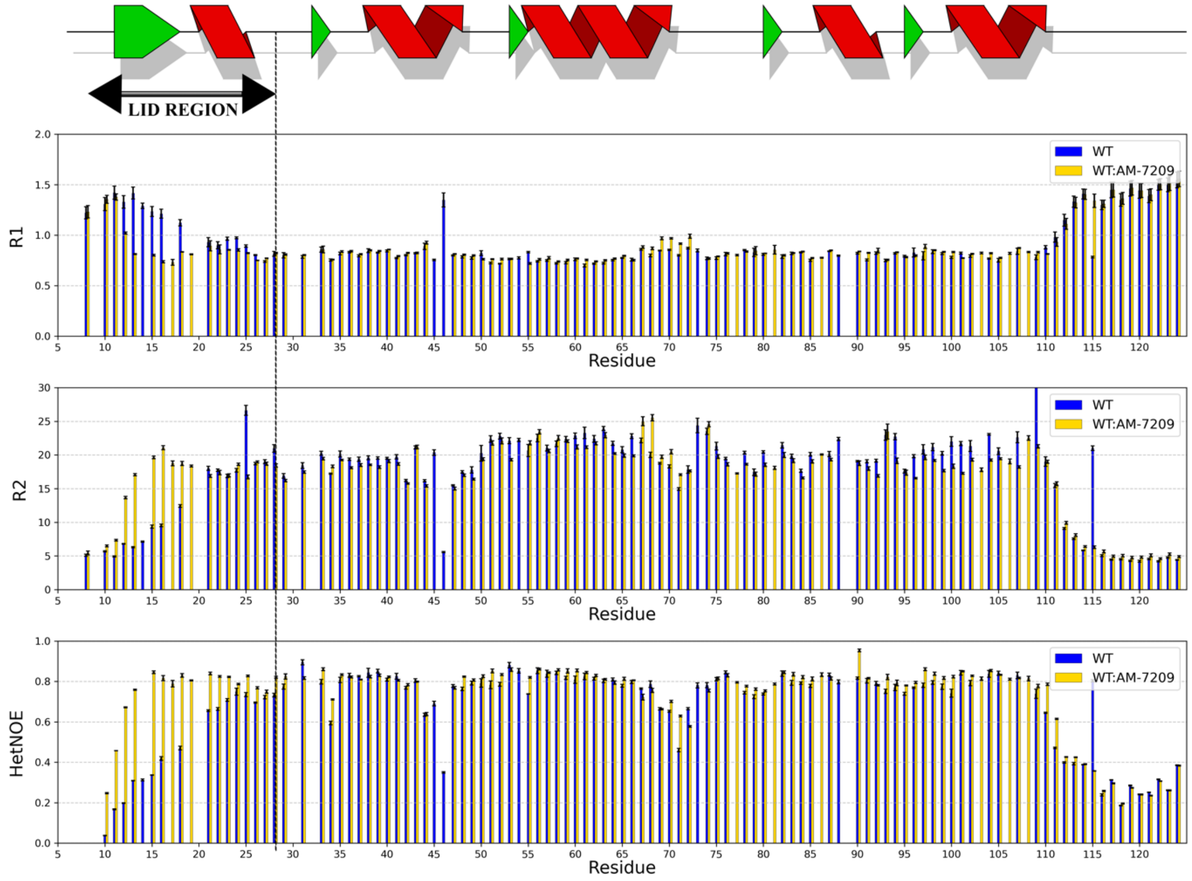

Fig. S18. Comparison of NMR backbone dynamics for WT in Apo (WT) and AM7209-bound form (WT:AM-7209). ^{15}N NMR relaxation rates R_1, R_2, and HetNOE were measured at pH 7.0 and 288K. Elevated ^{15}N R_2 rates and larger HetNOE values for residues (10-25) for WT:AM-7209 complex indicates restricted localized mobility and

ordered structural nature for the "lid" region compared to Apo form. Most HetNOE values for residues (10-20) for WT in Apo form are below 0.5, clearly indicating the absence of ordered structure and restricted localized mobility in the pico- to nanosecond timescale.

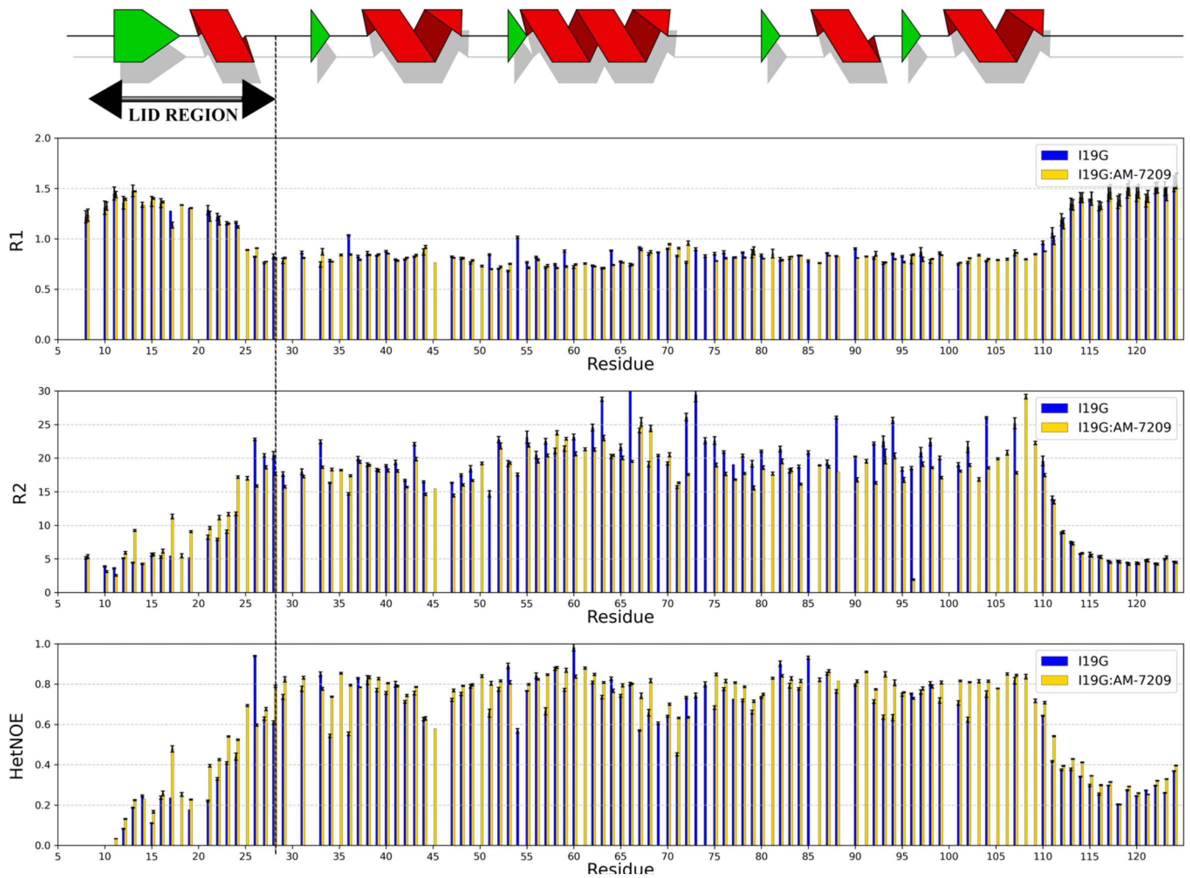

Fig. S19. Comparison of NMR backbone dynamics for I19G mutant in Apo and AM7209-bound form (I19G:AM-7209). NMR relaxation rates R_1, R_2, and HetNOE were measured at pH 7.0 and 288K. Similar R_1, R_2 relaxation rates and HetNOE profiles were observed for residues (10-25) from "lid" region for I19G:AM-7209 bound form relative to I19G mutant in Apo form.

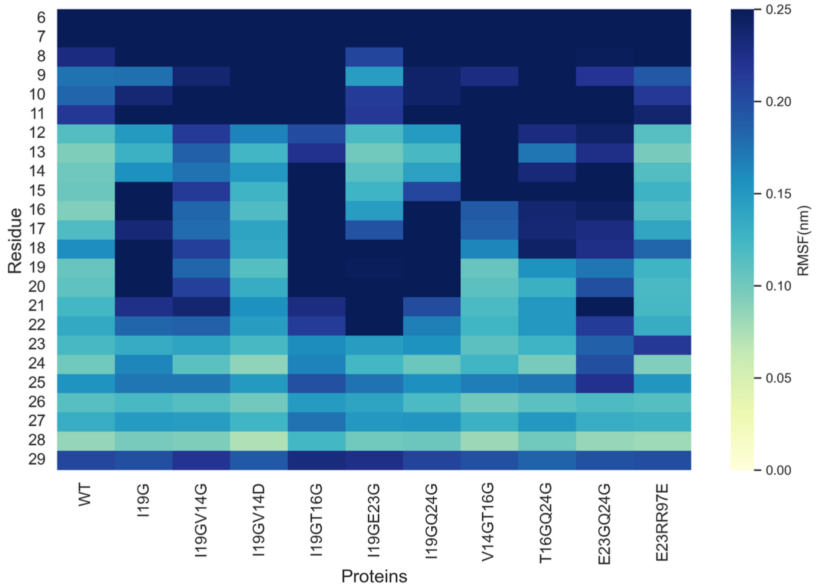

Fig. S20. RMSF calculations for double mutants. I19G and WT are included for reference.

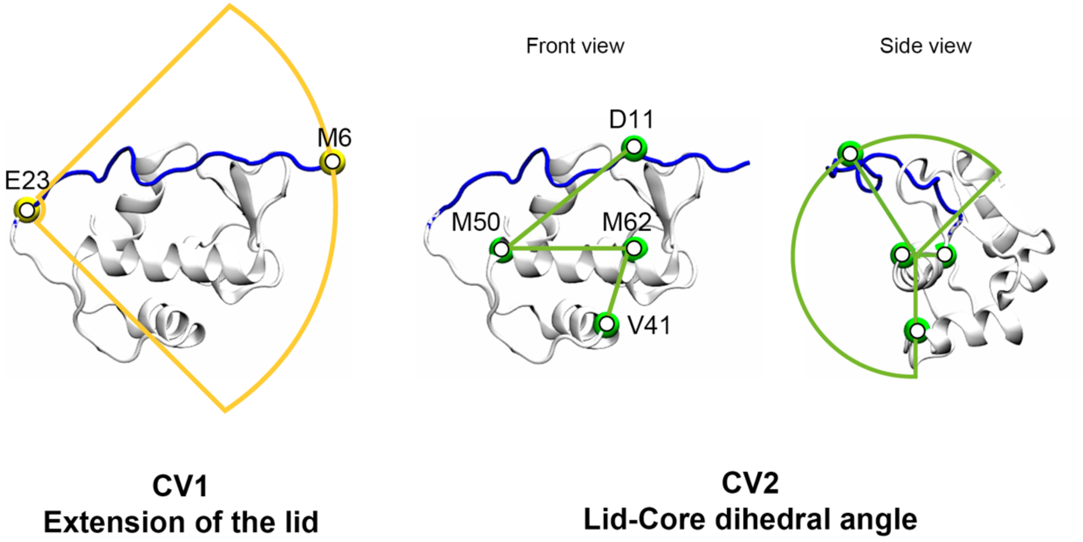

Fig. S21. Definition of the collective variable used to describe the conformational preferences of the MDM2 lid dynamics. MDM2 core (25-125) and lid (6-24) are shown in grey and blue respectively. The extension of the lid (CV1) is represented as the distance between the Cα atoms of residues M6 and E23 shown in yellow spheres. The lid-core dihedral angle (CV2) is the dihedral angle formed by the Cα atoms of residues D11, M50, M62, and V41, as shown in green spheres.

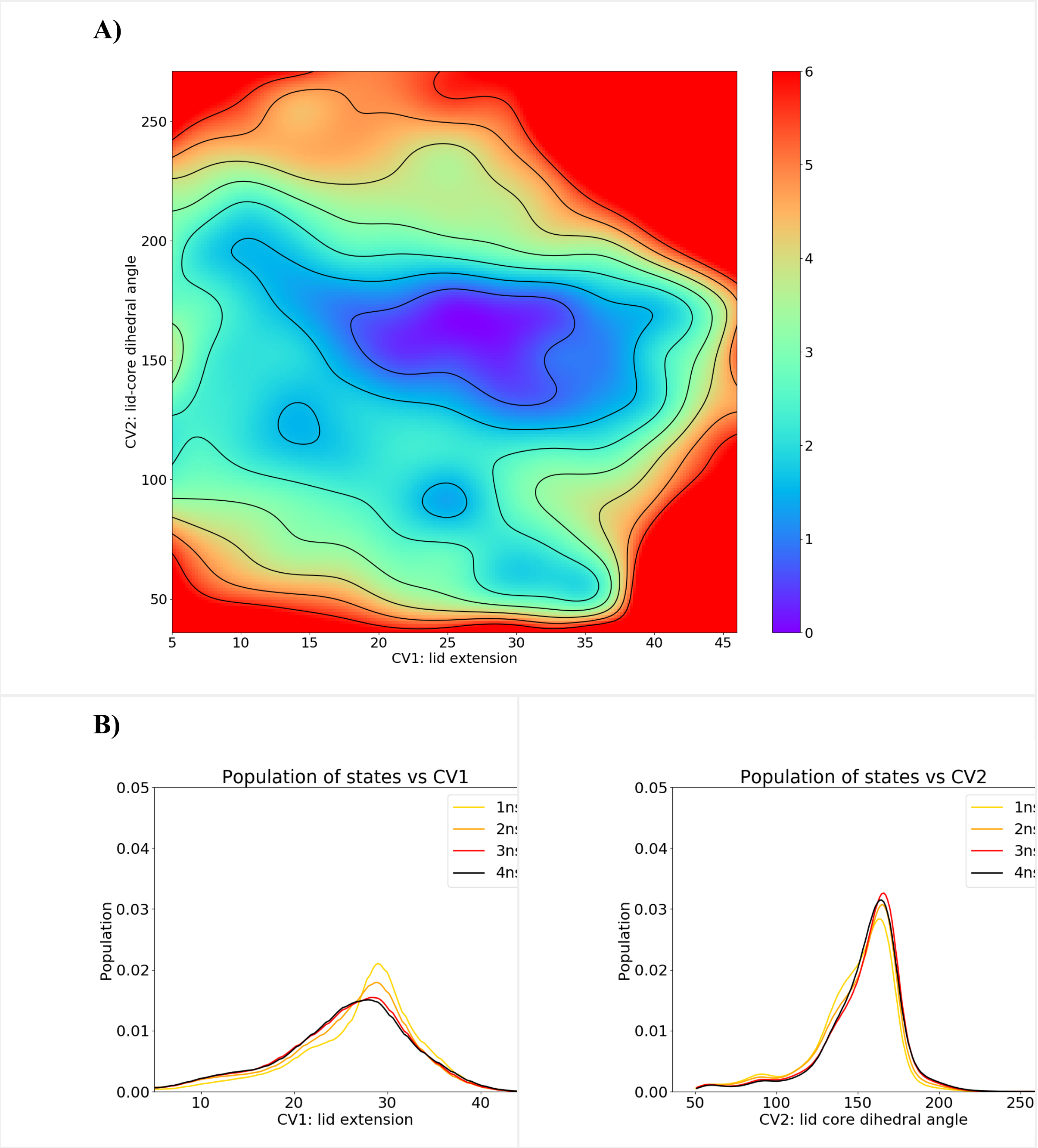

Fig. S22. FES and Convergence plots for MDM2-WT. A) FES of MDM2 (WT). Colorbar units are kcal/mol. B) Convergence plots along the two collective variables. Distribution of population along both collective variables at each ns of simulation.

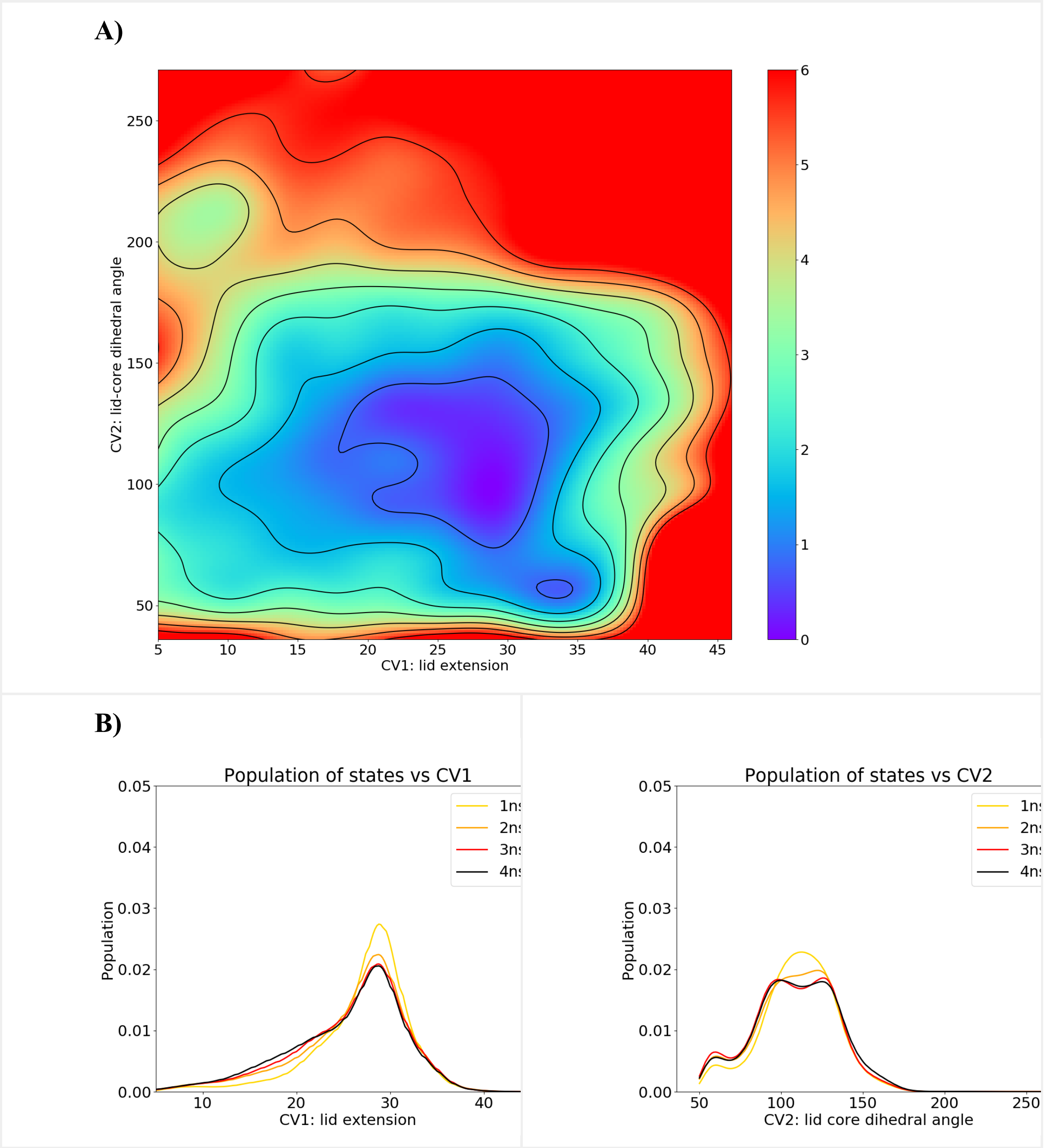

Fig. S23. FES and Convergence plots for MDM2-WT:AM7209. A) FES of the MDM2 bound to the AM7209 ligand (WT:AM7209). Colorbar units are kcal/mol. B) Convergence plots along the two collective variables. Distribution of population along both collective variables at each ns of simulation.

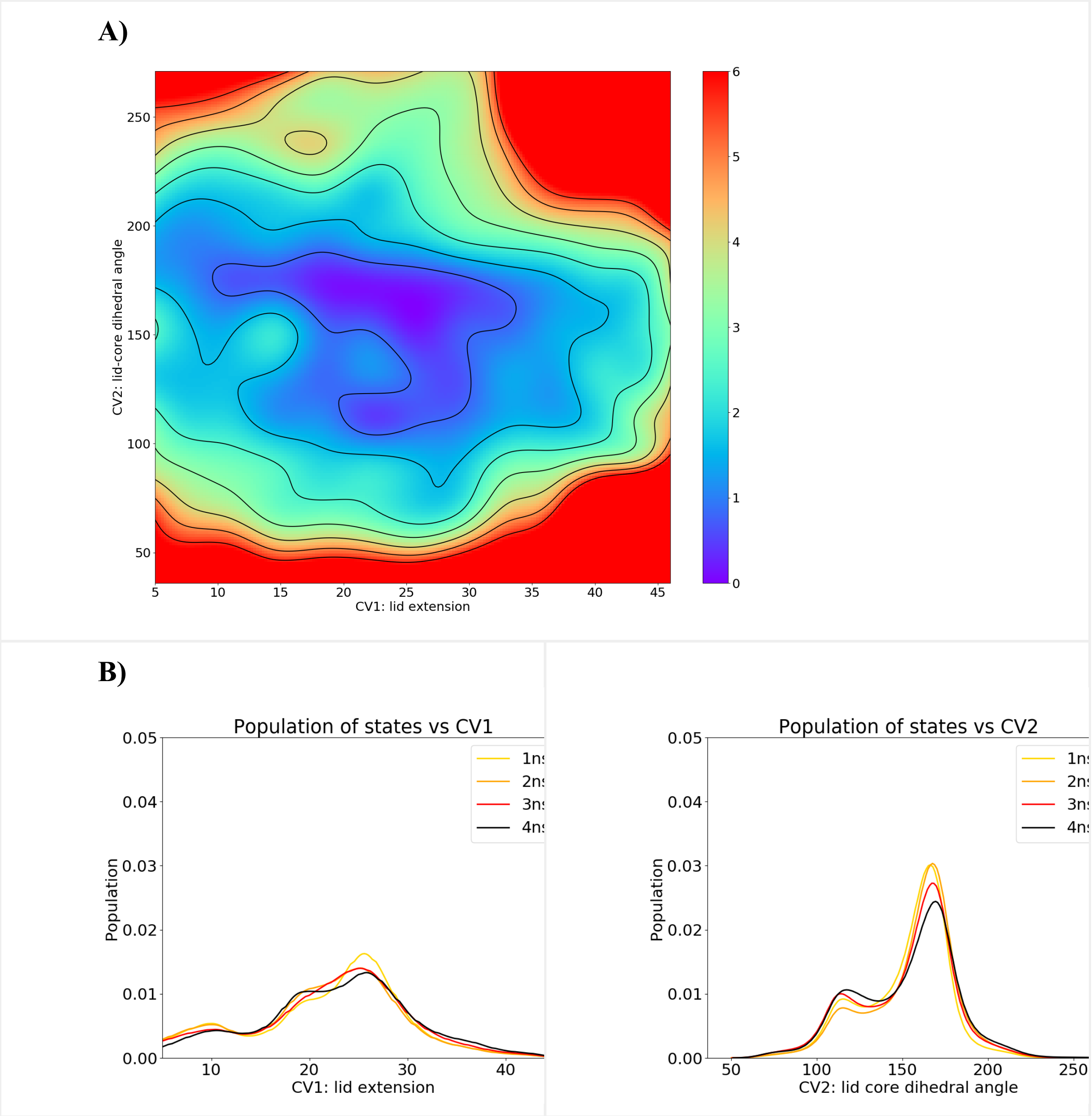

Fig. S24. FES and Convergence plots for MDM2-I19G. A) FES of the I19G MDM2 mutant (I19G). Colorbar units are kcal/mol. B) Convergence plots along the two collective variables. Distribution of population along both collective variables at each ns of simulation.

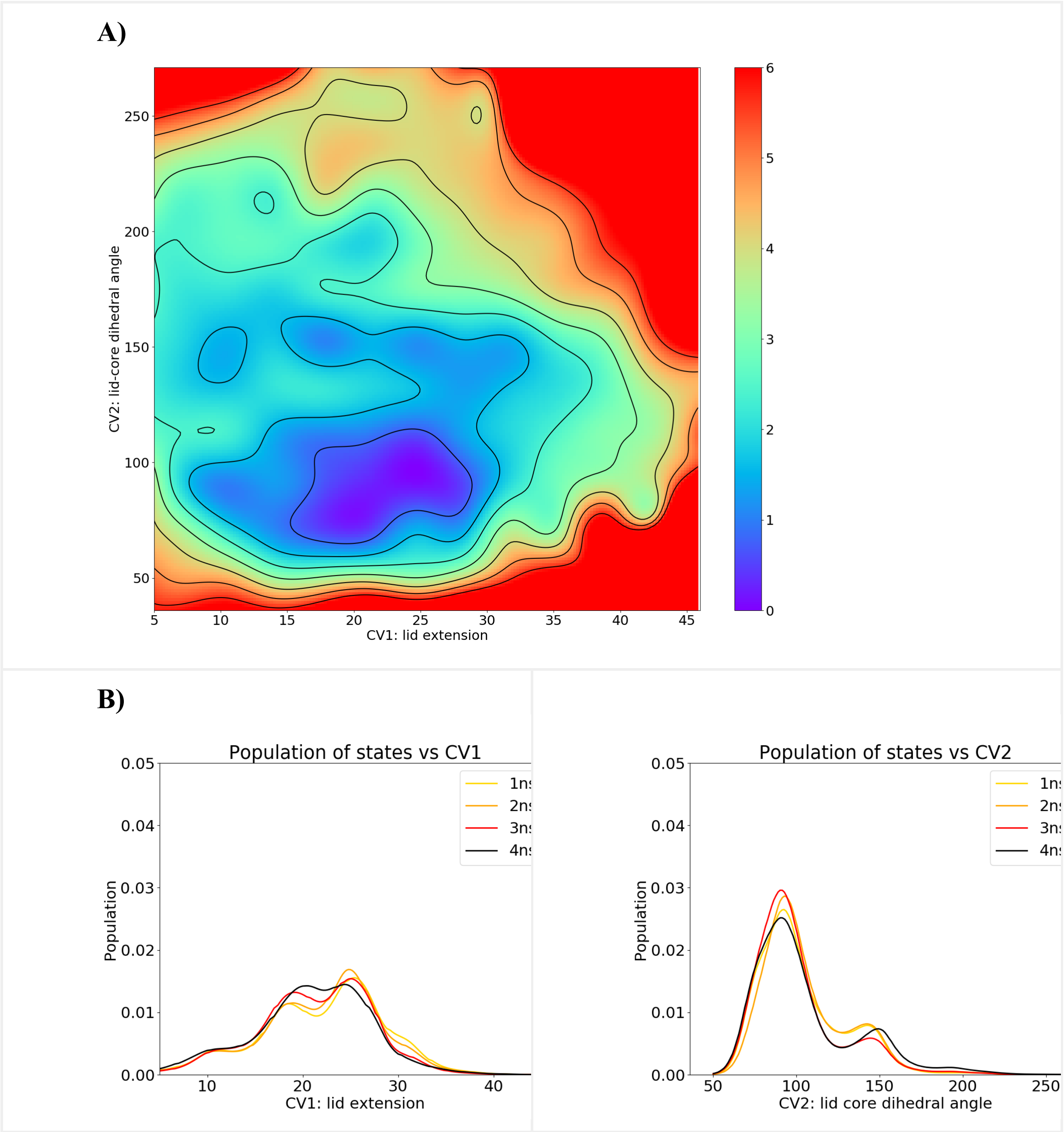

Fig. S25. FES and Convergence plots for MDM2-I19G:AM7209. A) FES of the I19G MDM2 mutant bound to the AM7209 ligand (I19G:AM7209). Colorbar units are kcal/mol. B) Convergence plots along the two collective variables. Distribution of population along both collective variables at each ns of simulation.

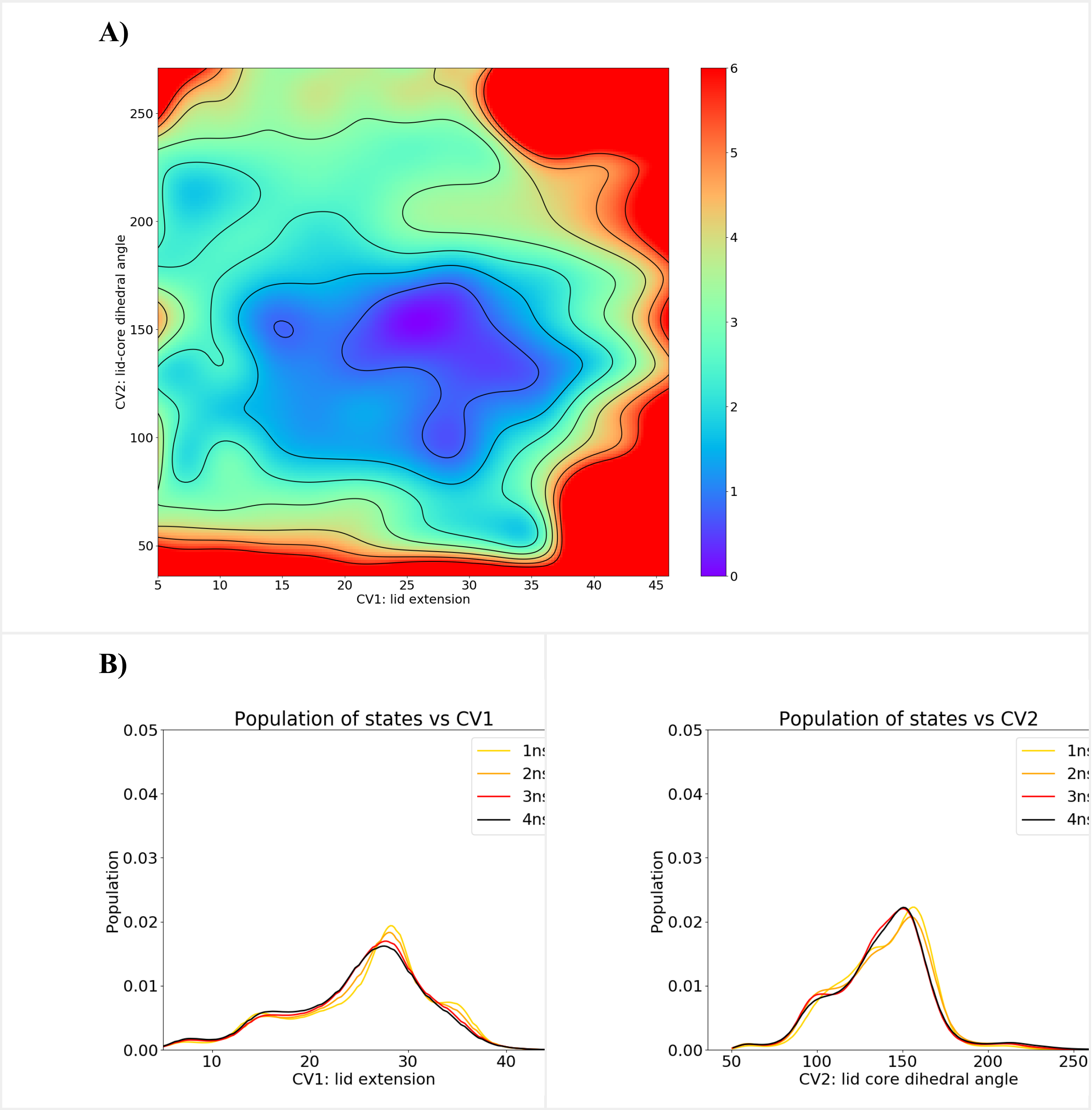

Fig. S26. FES and Convergence plots for MDM2-WT:AM7209-Cl. A) FES of the wt MDM2 bound to a variant of the AM7209 ligand (WT:AM7209-Cl). Colorbar units are kcal/mol. B) Convergence plots along the two collective variables. Distribution of population along both collective variables at each ns of simulation.

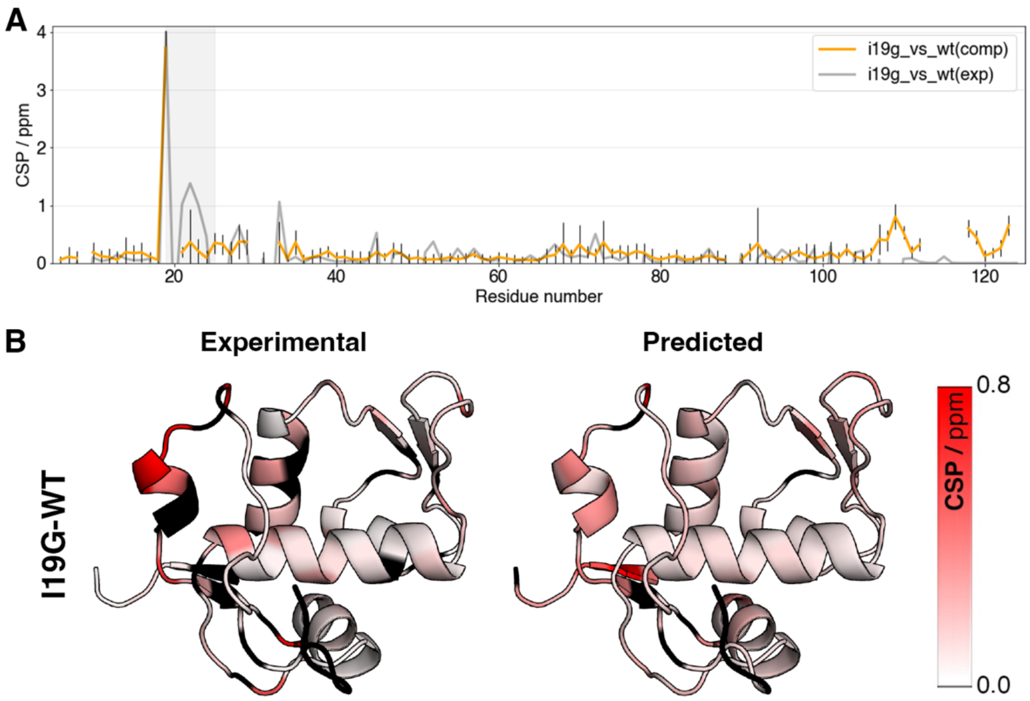

Fig. S27. Comparison between the experimental and predicted Chemical Shift Perturbation (CSP, ppm) between the I19G – WT proteins. A) Comparison of the NMR data versus the scaled SHIFTX2 prediction from the simulated apo conformational ensembles. B) Structure of the MDM2 protein in the lid ordered conformation colored by the experimental (left) and predicted CSP (right). The color bar shows the range of CSP values. Residues with no CSP are colored in black cartoon.

A

Conformational entropy of the apo and AM7209 bound MDM2 ensembles

PDB2ENTROPY

	-TΔS kcal/mol	-TΔΔS kcal/mol
**WT**	51,36 ± 1,47	
**WT:AM7209**	55,57 ± 1,60	4,21
**I19G**	49,76 ± 1,43	
**I19G:AM7209**	52,96 ± 1,49	3,20
		1,01

B

Translational and rotational entropy of AM7209 in the MDM2 complexes

PDB2TRENT

	-TΔS kcal/mol	-TΔΔS kcal/mol
**WT:AM7209**	2,01 ± 0,10	
**I19G:AM7209**	0,84 ± 0,01	1,18

C

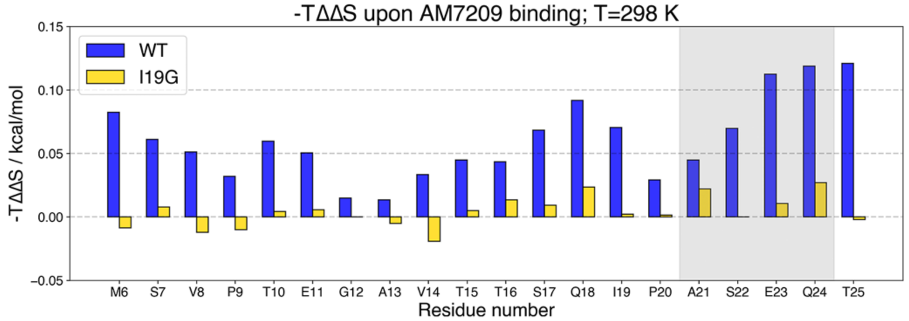

D

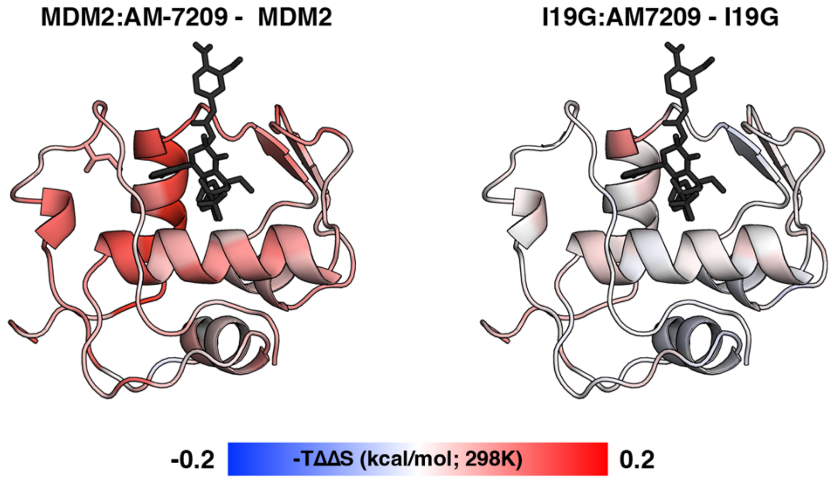

Fig S28. Analyses of the entropic changes in the conformational ensembles of the MDM2 systems. A) Data showing the values of -TΔS for each of the simulated systems and the -TΔΔS values comparing the AM7209 bound-unbound for the WT and the I19G mutant using PDB2ENTROPY. B) Data showing the translational and rotational entropies of the AM7209 ligand bound to the WT and the I19G proteins calculated with PDB2TRENT. C) Per residue decomposition of -TΔΔS values of the residues of the MDM2 lid comparing the AM7209 bound-unbound for the WT and the I19G mutant obtained using PDB2ENTROPY. D) Structure of the MDM2 protein in the lid ordered conformation colored by the entropic contribution of the conformational entropy of each residue of the protein (-TΔΔS at 298K, kcal/mol) as calculated using PDB2ENTROPY. The color bar shows the range of the -TΔΔS values along the protein structure. AM7209 is colored in black lines because its -TΔΔS value is not comparable between apo and holo systems.

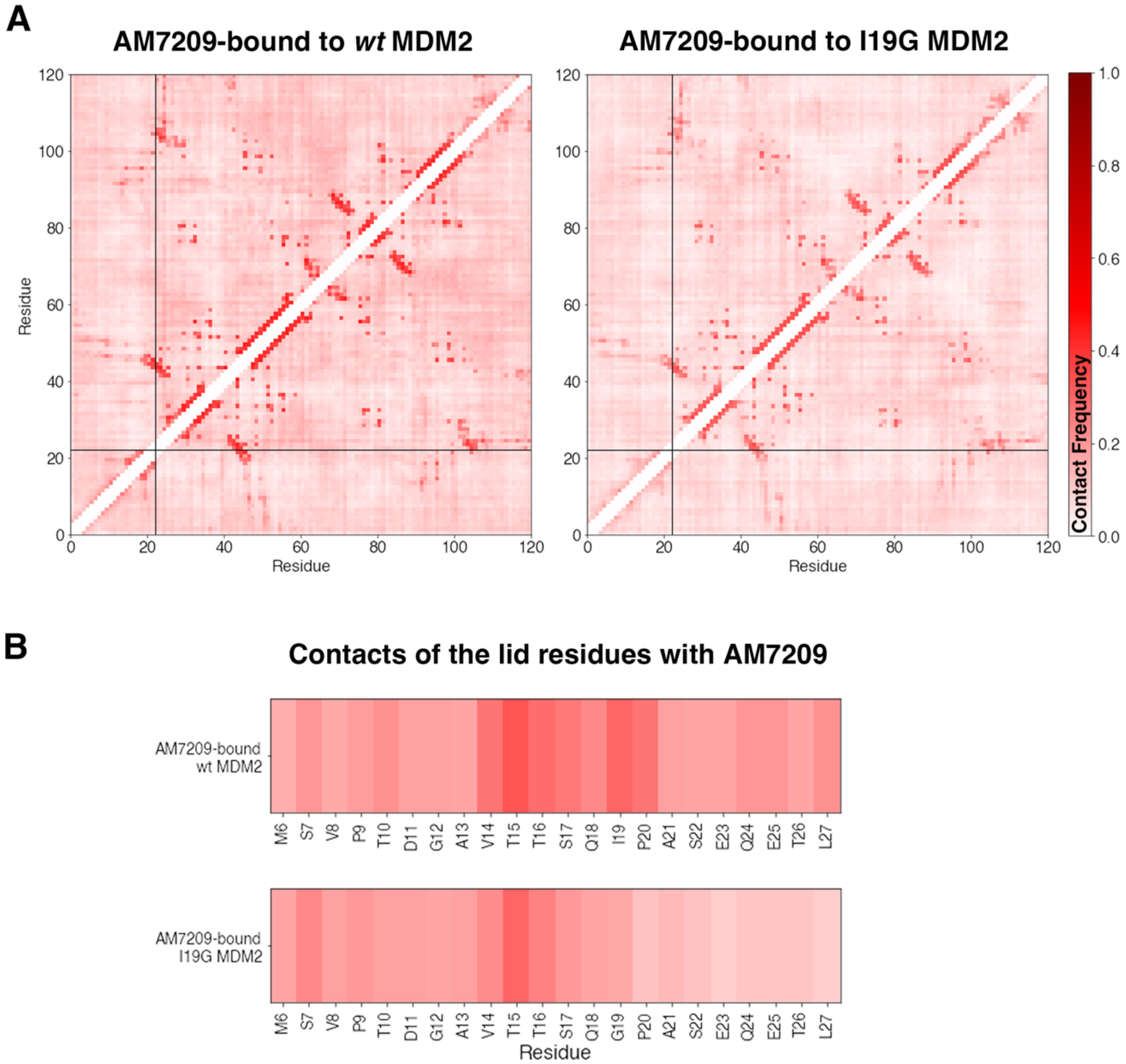

Fig S29. Contact maps of the AM7209 bound systems. A) Contact map of the AM7209 bound to the wildtype and I19G MDM2 proteins. B) Contact map between the AM7209 ligand and residues of the lid for the wildtype and the I19G MDM2 proteins. Colorbar indicates the Contact frequency between residues in all the panels, ranging from 0 to 1.

Tables

System	E_P	α_P	E_D	α_D
**WT**	-130310	10000	3000	78
**I19G-apo**	-130285	10000	3000	78
**WT-AM7209**	-123360	10000	3000	78
**I19G- AM-7209**	-123450	10000	3000	78

Table S1. Accelerated Molecular Dynamics parameters used in the present work.

Sample	Experiment	Spectral width (ppm)		Complex points			Offset (ppm)	
**WT**	^{15}N-HSQC	14.0 ×	26.0	2048×	256		4.757 ×	117.0
	^{13}C-HSQC (aliphatic)	14.0 ×	66.0	2048×	512		4.757 ×	38.0
	^{15}N-TOCSY HSQC[a,b]	12.0 ×	26.0 × 11.0	2048×	96×	256	4.757 ×	117.0× 4.757
	HNCO[a,c]	14.0 ×	26.0 × 15.0	2048×	96×	164	4.757 ×	117.0× 173.50
	HNCA[a,c]	14.0 ×	26.0 × 30.0	2048×	96×	80	4.757 ×	117.0× 55.0
	CBCA(CO)NH[a,c]	14.0 ×	26.0 × 66.0	2048×	96×	164	4.757 ×	117.0× 41.0
	HA(CO)NH[a,c]	14.0 ×	26.0 × 8.0	2048×	96×	196	4.757 ×	117.0× 4.757
**WT:AM-7209**	^{15}N-HSQC	14.0 ×	26.0	2048×	256		4.757 ×	117.0
	^{13}C-HSQC (aliphatic)	14.0 ×	66.0	2048×	512		4.757 ×	38.0
	HNCO[a,c]	14.0 ×	26.0 × 15.0	2048×	96×	164	4.757 ×	117.0× 173.50
	HN(CA)CO[a,c]	14.0 ×	26.0 × 15.0	2048×	96×	164	4.757 ×	117.0× 173.50
	HNCA[a,c]	14.0 ×	26.0 × 30.0	2048×	96×	80	4.757 ×	117.0× 55.0
	HN(CO)CA[a,c]	14.0 ×	26.0 × 30.0	2048×	96×	80	4.757 ×	117.0× 55.0
	HNCACB[a,c]	14.0 ×	26.0 × 66.0	2048×	96×	164	4.757 ×	117.0× 41.0
	CBCA(CO)NH[a,c]	14.0 ×	26.0 × 66.0	2048×	96×	164	4.757 ×	117.0× 41.0
	HA(CO)NH[a,c]	14.0 ×	26.0 × 8.0	2048×	96×	196	4.757 ×	117.0× 4.757
**I19G**	^{15}N-HSQC	14.0 ×	26.0	2048×	256		4.757 ×	117.0
	^{13}C-HSQC (aliphatic)	14.0 ×	66.0	2048×	512		4.757 ×	38.0
	HNCO[a,c]	14.0 ×	26.0 × 15.0	2048×	96×	164	4.757 ×	117.0× 173.50
	HN(CA)CO[a,c]	14.0 ×	26.0 × 15.0	2048×	96×	164	4.757 ×	117.0× 173.50
	HNCA[a,c]	14.0 ×	26.0 × 30.0	2048×	96×	80	4.757 ×	117.0× 55.0
	CBCA(CO)NH[a,c]	14.0 ×	26.0 × 66.0	2048×	96×	164	4.757 ×	117.0× 41.0
	HBHA(CO)NH[a,c]	14.0 ×	26.0 × 8.0	2048×	96×	196	4.757 ×	117.0× 4.757
**I19G:AM-7209**	^{15}N-HSQC	14.0 ×	26.0	2048×	256		4.757 ×	117.0
	^{13}C-HSQC (aliphatic)	14.0 ×	66.0	2048×	512		4.757 ×	38.0
	HNCO[a,c]	14.0 ×	26.0 × 15.0	2048×	96×	164	4.757 ×	117.0× 173.50
	HN(CA)CO[a,c]	14.0 ×	26.0 × 15.0	2048×	96×	164	4.757 ×	117.0× 173.50
	HNCA[a,c]	14.0 ×	26.0 × 30.0	2048×	96×	80	4.757 ×	117.0× 55.0
	HN(CO)CA[a,c]	14.0 ×	26.0 × 30.0	2048×	96×	80	4.757 ×	117.0× 55.0
	HNCACB[a,c]	14.0 ×	26.0 × 66.0	2048×	96×	164	4.757 ×	117.0× 41.0
	CBCA(CO)NH[a,c]	14.0 ×	26.0 × 66.0	2048×	96×	164	4.757 ×	117.0× 41.0
	HBHA(CO)NH[a,c]	14.0 ×	26.0 × 8.0	2048×	96×	196	4.757 ×	117.0× 4.757

[a] Non-uniform sampling density, 25% of data matrix recorded

[b] Indirect (F1, ^{1}H) × indirect (F2, ^{15}N) × direct (F3, ^{1}H)

[c] Indirect (F1,^{13}C) × indirect (F2, ^{15}N) × direct (F3, ^{1}H)

Table S2. List of 2D and 3D spectra used to assign the WT, WT:AM-7209, I19G and I19G:AM-7209 (spectral width; complex points and offset are indicated.)

Sample	Experiment	Scans	Spectral width (ppm)	Complex points	Time Points
**WT**	**^{15}N $\boldsymbol{R_1}$**	16	14.0 × 26.0	2048 × 256	0.05;0.25(twice), 0.50; 0.75;1.00;1.25;1.50 (s)
	**^{15}N $\boldsymbol{R_2}$**	16	14.0 × 26.0	2048 × 256	16.96 (twice), 33.92; 50.88;67.84;84.80;118.72;152.64;186.56 (ms)
	**{^{1}H}-^{15}N NOE**	64	14.0 × 26.0	2048 × 256	-
**WT: AM7209**	**^{15}N $\boldsymbol{R_1}$**	16	14.0 × 26.0	2048 × 256	0.05;0.25(twice), 0.50; 0.75;1.00;1.25;1.50 (s)
	**^{15}N $\boldsymbol{R_2}$**	16	14.0 × 26.0	2048 × 256	16.96 (twice), 33.92; 50.88;67.84;84.80;118.72;152.64;186.56 (ms)
	**{^{1}H}-^{15}N NOE**	64	14.0 × 26.0	2048 × 256	-
**I19G**	**^{15}N $\boldsymbol{R_1}$**	32	14.0 × 26.0	2048 × 256	0.05;0.25(twice), 0.50; 0.75;1.00;1.25;1.50 (s)
	**^{15}N $\boldsymbol{R_2}$**	32	14.0 × 26.0	2048 × 256	16.96 (twice), 33.92; 50.88;67.84;84.80;118.72;152.64;186.56 (ms)
	**{^{1}H}-^{15}N NOE**	64	14.0 × 26.0	2048 × 256	-
**I19G: AM-7209**	**^{15}N $\boldsymbol{R_1}$**	16	14.0 × 26.0	2048 × 256	0.05;0.25(twice), 0.50; 0.75;1.00;1.25;1.50 (s)
	**^{15}N $\boldsymbol{R_2}$**	16	14.0 × 26.0	2048 × 256	16.96 (twice), 33.92; 50.88;67.84;84.80;118.72;152.64;186.56 (ms)
	**{^{1}H}-^{15}N NOE**	64	14.0 × 26.0	2048 × 256	-

Table S3. Parameters used in NMR backbone dynamics measurements

	Hydrophobic SASA of the protein				
	SASA	Standard deviation	Difference (unbound-bound)		Standard deviation
	Å^2	Å^2	Å^2	%	%
WT	2020	174			
WT:AM-7209	1839	177	181	9.0	1.0
I19G	2063	181			
I19G:AM-7209	1908	174	155	7.5	0.8
	Total SASA of the AM-7209 ligand				
	SASA	Standard deviation	Difference (unbound-bound)		Standard deviation
	Å^2	Å^2	Å^2	%	%
Unbound	1005	26			
WT:AM-7209	272	51	733	73.0	0.4
Unbound	1005	26			
I19G:AM-7209	329	71	676	67.2	1.0

Table S4. Solvent Accessible Surface Area (SASA) of the conformational ensembles of MDM2. (Top) Data showing the difference in hydrophobic SASA of the protein. Bottom) Data showing the difference in SASA of the AM-7209 ligand upon binding to the WT and I19G mutant proteins.

System name	# Simulations	Simulation box dimensions	# of atoms	# Water molecules	# Cl$^-$ Ions
wt	**unbiased MD** 3*100ns (300 ns)<br><br>**aMD** 3*100ns (300 ns)<br><br>**US** 524*4ns (2096 ns)<br><br>**Total:** 2696 ns per system	~75x75x75 Å	43211	13751	3 (neutralising conditions)
wt+ am7209		~75x74x75 Å	41891	13282	2 (neutralising conditions)
wt+ amg-cl		~75x74x75 Å	41893	13282	2 (neutralising conditions)
i19g		~74x74x74 Å	40688	12914	3 (neutralising conditions)
i19g+ am7209		~74x74x75 Å	40969	12978	2 (neutralising conditions)

Table S5. Details of the simulations included in the aMD/US/vFEP simulations included in this work.

Datasets

Dataset S1. (lid ensembles).

Lid conformational ensembles sampled from the Free Energy Surfaces of MDM2 WT and I19G, in free and AM-7209 bound forms. These datasets are available at https://github.com/michellab/MDM2-D2O-paper .

Dataset S2. (chemical shifts).

Chemical shifts measurements for MDM2 WT and I19G in free and AM-7209 bound forms. These datasets are available at https://github.com/michellab/MDM2-D2O-paper .

SI References

(1) Loeffler, Hannes H.; Michel J.; Woods C. FESetup: Automating Setup for Alchemical Free Energy Simulations, *J. Chem. Inf. Model*. **2015**, 55 (12), 2485–2490. DOI: 10.1021/acs.jcim.5b00368.

(2) Bueren-Calabuig JA; Michel J. Elucidation of Ligand-Dependent Modulation of Disorder-Order Transitions in the Oncoprotein MDM2. *PLOS Computational Biology.* **2015**, 11(6): e1004282. DOI: 10.1371/journal.pcbi.1004282.

(3) Rew, Y.; Sun, D. Q.; Yan, X. L.; Beck, H. P.; Canon, J.; Chen, A.; Duquette, J.; Eksterowicz, J.; Fox, B. M.; Fu, J. S.; et al. Discovery of AM-7209, a Potent and Selective 4-Amidobenzoic Acid Inhibitor of the MDM2-p53 Interaction. *Journal of Medicinal Chemistry.* **2014**, 57 (24), 10499-10511. DOI: 10.1021/jm501550p; Vassilev L. T., Vu B.T., Graves B., Carvajal D., Podlaski F., Filipovic Z., Kong N., Kammlott U., Lukacs C., Klein C., Fotouhi N. and Liu E. A., In Vivo Activation of the p53 Pathway by Small-Molecule Antagonists of MDM2 Science, **2004**, (303), 844–848.

(4) Abraham M.J.; Murtola T.; Schulz R.; Páll S.; Smith J.C.; Hess B.; Lindahl E. GROMACS: High performance molecular simulations through multi-level parallelism from laptops to supercomputers. *SoftwareX.* **2015**, 1–2, 19–25. DOI: 10.1016/j.softx.2015.06.00.

(5) Jorgensen, W. L.; Chandrasekhar, J.; Madura, J. D.; Impey, R. W.; Klein, M. L. Comparison of Simple Potential Functions for Simulating Liquid Water. *J. Chem. Phys.* **1983**, *79* (2), 926–935. DOI:10.1063/1.445869.

(6) Li, D.-W.; Brüschweiler, R. NMR-Based Protein Potentials. *Angew. Chemie Int. Ed.* **2010**, *49* (38), 6778–6780. DOI: 10.1002/anie.201001898.

(7) Joung, I. S.; Cheatham, T. E. Determination of Alkali and Halide Monovalent Ion Parameters for Use in Explicitly Solvated Biomolecular Simulations. *J. Phys. Chem. B.* **2008**, *112* (30), 9020–9041. DOI: 10.1021/jp8001614.

(8) Wang, J.; Wolf, R. M.; Caldwell, J. W.; Kollman, P. A.; Case, D. A. Development and Testing of a General Amber Force Field. *J. Comput. Chem.* **2004**, *25* (9), 1157–1174. DOI: 10.1002/jcc.20035.

(9) Wang, J.; Wang, W.; Kollman, P. A.; Case, D. A. Automatic Atom Type and Bond Type Perception in Molecular Mechanical Calculations. *J. Mol. Graph. Model.* **2006**, *25* (2), 247–260. DOI: 10.1016/j.jmgm.2005.12.005.

(10) Case, D. A.; Betz, R. M.; Cerutti, D. S.; Cheatham III, T. E.; Darden, T. A.; Duke, R. E.; Giese, T. J.; Gohlke, H.; Goetz, A. W.; Homeyer, N.; Izadi, S.; Janowski, P.; Kaus, J.; Kovalenko, A.; Lee, T. S.; LeGrand, S.; Li, P.; Lin, C.; Luchko, T.; Luo, R.; Madej, B.; Mermelstein, D.; Merz, K. M.; Monard, G.; Nguyen, H.; Nguyen, H. T.; Omelyan, I.; Onufriev, A.; Roe, D. R.; Roitberg, A.; Sagui, C.; Simmerling, C. L.; Botello-Smith, W. M.; Swails, J.; Walker, R. C.; Wang, J.; Wolf, R. M.; Wu, X.; Xiao, L.; Kollman, P. A. Amber 16. *University of California, San Francisco*. **2016**. DOI: 10.1002/jcc.23031.

(11) Ryckaert , J.-P.; Ciccotti G.; Berendsen, H.J.C. Numerical integration of the cartesian equations of motion of a system with constraints: molecular dynamics of n-alkanes. *J. Comput. Phys.* **1977**, 23, p. DOI: 10.1016/j.cpc.2008.10.020.

(12) Pierce, L. C. T.; Salomon-Ferrer, R.; Augusto F. De Oliveira, C.; McCammon, J. A.; Walker, R. C. Routine Access to Millisecond Time Scale Events with Accelerated Molecular Dynamics. *J. Chem. Theory Comput.* **2012**, *8* (9), 2997–3002. DOI: 10.1021/ct300284c.

(13) Mendoza-Martinez, C.; Papadourakis , M.; Llabrés S.; Gupta, Arun A.; Barlow, P. N.; Michel J. Energetics of a protein disorder–order transition in small molecule recognition. *Chem. Sci.* **2022**,13, 5220-5229. DOI: 10.1039/D2SC00028H.

(14) Lee, T.-S.; Radak, B. K.; Pabis, A.; York, D. M. A New Maximum Likelihood Approach for Free Energy Profile Construction from Molecular Simulations. *J. Chem. Theory Comput.* **2013**, *9* (1), 153–164. DOI: 10.1021/ct300703z.

(15) Roe, D. R.; Cheatham III, T. E. A Parallelization of CPPTRAJ enables large scale analysis of molecular dynamics trajectory data *J. Comput. Chem.* **2018**, 3*9* (25), 2110-2117. DOI: 10.1002/jcc25382.

(16) McGibbon, R. T. ; Beauchamp, K. A. ; Harrigan, M. P. ; Klein, C. ; Swails, J. M. ; Hernandez, C. X. ; Schwantes, C. R. ; Wang, L. ; Lanes, T. J. ; Pande, V. S. MDTraj: A Modern Open Library for the Analysis of Molecular Dynamics Trajectories *Biophys. J.* **2015**, *109 (8),* 1528-1532, DOI: 10.1016:j.bpj.2015.08.015

(17) Han, B. ; Liu, Y. ; Ginzinger, S. W.; Wishard, D. S. SHIFTX2: significantly improved chemical shift prediction *J. Biomol. NMR* **2011**, *50 (1)*, 43-57, DOI: 10.1007: s10858-011-9478-4

(18) Juarez-Jimenez et al. Dynamic design: manipulation of millisecond timescale motions on the energy landscape of cyclophilin A *Chem. Sci.* **2020**, 11, 2670-2680, DOI:10.1039/C9SC04696H

(19) Fogolorai, F. ; Maloku, O. ; Foumthuim, C. J. D. ; Corazza, A. ; Esposito G. PDB2ENTROPY and PDB2TRENT: conformational and translational-rotational entropy from molecular ensembles *J. Chem. Inf. Model.* **2018**, *58(7)*, 1319-1324, DOI:10.1021/acs.jcim.8b00143

(20) Cai M.; Huang Y.; Sakaguchi K.; Clore G.M.; Gronenborn A. M.; Craigie R. An efficient and cost-effective isotope labeling protocol for proteins expressed in Escherichia coli. *Journal of Biomolecular NMR.* **1998**, *11* (1), 97-102, journal article. DOI: 10.1023/a:1008222131470.

(21) Sigurskjold, B. W. Exact Analysis of Competition Ligand Binding by Displacement Isothermal Titration Calorimetry. *Analytical Biochemistry.* **2000**, *277* (2), 260-266. DOI: 10.1006/abio.1999.4402.
(22) Sattler, M., Schleucher J.; Griesinger C. Heteronuclear multidimensional NMR experiments for the structure determination of proteins in solution employing pulsed field gradients. *Spectroscopy.* **1999**, *34* (2), 93-158. DOI: 10.1016/S0079-6565(98)00025-9.
(23) Michelsen K.; Jordan J. B.; Lewis J.; Long A. M.; Yang E.; Rew Y.; Zhou J.; Yakowec P.; Schnier P. D.; Huang X.; Poppe L. Ordering of the N-Terminus of Human MDM2 by Small Molecule Inhibitors. *Journal of the American Chemical Society*. **2012**, 134(41), 17059-17067. DOI: 10.1021/ja305839b.
(24) Ying, J.; Delaglio F.; Torchia, D. A.; Bax, A. Sparse multidimensional iterative lineshape-enhanced (SMILE) reconstruction of both non-uniformly sampled and conventional NMR data. *Journal of Biomolecular NMR.* **2017**, *68* (2), 101-118. DOI: 10.1007/s10858-016-0072-7.
(25) Delaglio, F.;, Grzesiek, S.; Vuister G. W.; Zhu G.; Pfeifer J.; Bax, A. NMRPipe: a multidimensional spectral processing system based on UNIX pipes. *Journal of Biomolecular NMR.* **1995**, *6* (3), 277-293. DOI: 10.1007/bf00197809.
(26) Lee, W.; Tonelli M.; Markley, J. L. NMRFAM-SPARKY: enhanced software for biomolecular NMR spectroscopy. *Bioinformatics.* **2015**, *31* (8), 1325-1327. DOI: 10.1093/bioinformatics/btu830.
(27) Lee, W.; Rahimi M.; Lee, Y.; Chiu, A. POKY: a software suite for multidimensional NMR and 3D structure calculation of biomolecules. *Bioinformatics.* **2021**, *37* (18), 3041-3042. DOI: 10.1093/bioinformatics/btab180.
(28) Zimmerman, D. E.; Kulikowski C. A.; Huang Y.; Feng W.; Tashiro M.; Shimotakahara S.; Chien C.; Powers, R.; Montelione, G. T. Automated Analysis of Protein NMR Assignments Using Methods from Artificial Intelligence. *Journal of Molecular Biology.* **1997**, *269* (4), 592-610. DOI: 10.1006/jmbi.1997.1052.
(29) Lee, W.; Markley, J. L. PINE-SPARKY.2 for automated NMR-based protein structure research. *Bioinformatics,* **2018**, *34* (9), 1586-1588. DOI: 10.1093/bioinformatics/btx785
(30) Maciejewski, M. W.; 1 , Schuyler, A. D.; Gryk M. R.; Moraru, I. I.; Romero, P. R.; Ulrich , E. L.; Eghbalnia, H. R.; 3 , Livny, M.; Delaglio , F.; Hoch, J. C. NMRbox: A Resource for Biomolecular NMR Computation. *Biophysical Journal.* **2017**, *112* (8), 1529-1534. DOI: 10.1016/j.bpj.2017.03.011.
(31) Mulder, F. A. ; Schipper D.; Bott, R.; Boelens, R. Altered flexibility in the substrate-binding site of related native and engineered high-alkaline Bacillus subtilisins. *Journal of Molecular Biology. 1999*, 292(1), 111-123. DOI: 10.1006/jmbi.1999.3034
(32) Uhrinova S.; Uhrin D.; Powers H.; Watt K.; Zheleva D.; Fischer P.;, McInnes C.; Barlow P. N. Structure of Free MDM2 N-terminal Domain Reveals Conformational Adjustments that Accompany p53-binding. *Journal of Molecular Biology*. **2005**, 350(3), 587-598. DOI: 10.1016/j.jmb.2005.05.010.
(33) Ahlner A.; Carlsson M.; Jonsson, B.-H.; Lundström, P. PINT: a software for integration of peak volumes and extraction of relaxation rates. *Journal of Biomolecular NMR.* **2013**, *56* (3), 191-202. DOI: 10.1007/s10858-013-9737-7.
